# Adaptive Changes in Group 2 Metabotropic Glutamate Receptors Underlie the Deficit in Recognition Memory Induced by Methamphetamine in Mice

**DOI:** 10.1523/ENEURO.0523-23.2024

**Published:** 2024-08-02

**Authors:** Carla Letizia Busceti, Luisa Di Menna, Sonia Castaldi, Giovanna D’Errico, Alice Taddeucci, Valeria Bruno, Francesco Fornai, Anna Pittaluga, Giuseppe Battaglia, Ferdinando Nicoletti

**Affiliations:** ^1^Department of Molecular Pathology, IRCCS Neuromed, Pozzilli 86077, Italy; ^2^Department of Physiology and Pharmacology, University Sapienza, Roma 00185, Italy; ^3^Department of Pharmacy, University of Genova, Genova 16148, Italy; ^4^Department of Translational Research and New Technologies in Medicine and Surgery, University of Pisa, Pisa 56126, Italy; ^5^IRCCS Ospedale Policlinico San Martino, Genova 16145, Italy

**Keywords:** AGS3, cognitive dysfunction, glutamate release, metabotropic glutamate receptors, prefrontal cortex, Rab3A

## Abstract

Cognitive dysfunction is associated with methamphetamine use disorder (MUD). Here, we used genetic and pharmacological approaches to examine the involvement of either Group 2 metabotropic glutamate (mGlu2) or mGlu3 receptors in memory deficit induced by methamphetamine in mice. Methamphetamine treatment (1 mg/kg, i.p., once a day for 5 d followed by 7 d of withdrawal) caused an impaired performance in the novel object recognition test in wild-type mice, but not in mGlu2^−/−^ or mGlu3^−/−^ mice. Memory deficit in wild-type mice challenged with methamphetamine was corrected by systemic treatment with selectively negative allosteric modulators of mGlu2 or mGlu3 receptors (compounds VU6001966 and VU0650786, respectively). Methamphetamine treatment in wild-type mice caused large increases in levels of mGlu2/3 receptors, the Type 3 activator of G-protein signaling (AGS3), Rab3A, and the vesicular glutamate transporter, vGlut1, in the prefrontal cortex (PFC). Methamphetamine did not alter mGlu2/3-mediated inhibition of cAMP formation but abolished the ability of postsynaptic mGlu3 receptors to boost mGlu5 receptor-mediated inositol phospholipid hydrolysis in PFC slices. Remarkably, activation of presynaptic mGlu2/3 receptors did not inhibit but rather amplified depolarization-induced [^3^H]-D-aspartate release in synaptosomes prepared from the PFC of methamphetamine-treated mice. These findings demonstrate that exposure to methamphetamine causes changes in the expression and function of mGlu2 and mGlu3 receptors, which might alter excitatory synaptic transmission in the PFC and raise the attractive possibility that selective inhibitors of mGlu2 or mGlu3 receptors (or both) may be used to improve cognitive dysfunction in individuals affected by MUD.

## Significance Statement

Targeting cognitive dysfunction may reduce methamphetamine craving and relapse in individuals who use methamphetamine. Using the novel object recognition test for the study of recognition memory, we found that cognitive impairment caused by methamphetamine in mice was corrected by genetic deletion or selective pharmacological blockade of either Group 2 metabotropic glutamate (mGlu2) or mGlu3 receptors, two mGlu receptor subtypes that control synaptic activity by restraining glutamate release. Interestingly, mGlu2/3 receptors were upregulated in the prefrontal cortex of methamphetamine-treated mice and showed an inverse mode of operation by enhancing depolarization-induced glutamate release. These findings suggest that selective mGlu2 or mGlu3 receptor antagonists may improve cognitive function in individuals affected by methamphetamine use disorder.

## Introduction

Individuals affected by methamphetamine use disorder (MUD) show impairments in different domains of cognitive function, such as learning and memory, executive function information processing, and attention tasks (Ersche et al., 2006; Scott et al., 2007; Rendell et al., 2009; Harlé et al., 2015).

Cognitive deficit persists during abstinence and may have a strong impact on compulsive behavior and resistance to relapsing in individuals who use methamphetamine (Kalechstein et al., 2003; Simon et al., 2004, 2010; Kim et al., 2005; Paulus et al., 2005; Gowin et al., 2014; Liang et al., 2018). Hence, understanding the molecular mechanisms underlying methamphetamine-induced cognitive dysfunction may shed new lights into the pathophysiology of MUD and pave the way to novel pharmacological treatments.

Group 2 metabotropic glutamate receptors (mGlu2 and mGlu3 receptors) are coupled to G_i/o_ proteins and regulate synaptic transmission and plasticity acting at different levels of the tripartite synapse (Nicoletti et al., 2011). Both receptors are localized in the preterminal region of presynaptic terminals, where their activation negatively regulates neurotransmitter release. In addition, mGlu3 receptors have also been found in postsynaptic elements, and their activation boosts mGlu5 receptor signaling (Di Menna et al., 2018; Joffe et al., 2019; Dogra et al., 2021).

The mGlu2 receptor in particular has been linked to the pathophysiology of substance use disorder, and pharmacological activation of mGlu2 receptors reduces self-administration and cue-induced reinstatement of psychostimulants (Caprioli et al., 2018). Peter Kalivas and his associates have demonstrated that vulnerability to drug relapse is associated with a bidirectional impairment of activity-dependent synaptic plasticity at the synapses between nerve terminals of cortical pyramidal neurons and medium spiny neurons of the core of the nucleus accumbens (NAc). This defect is caused, in part, by an impaired activation of presynaptic mGlu2/3 receptors (Moussawi et al., 2009; Moussawi and Kalivas, 2010). Selective pharmacological activation of mGlu2 receptors reduced cue-induced methamphetamine seeking in abstinent rats (Caprioli et al., 2015).

We found that genetic deletion of either mGlu2 or mGlu3 receptors affects the primary mechanism of action of methamphetamine, as well as a number of behavioral tasks related to methamphetamine addiction. For example, mGlu2 receptor knock-out (mGlu2^−/−^) mice showed reductions in methamphetamine-stimulated dopamine release in striatal slices, conditioned place preference (CPP) to methamphetamine, and methamphetamine-induced increase in motor activity, whereas mGlu3^−/−^ mice showed increases in CPP to methamphetamine and motor sensitization to methamphetamine (Busceti et al., 2021). These findings contrast with the evidence that pharmacological activation of mGlu2 receptors enhances alcohol, cocaine, and nicotine seeking (reviewed by Caprioli et al., 2018), suggesting that the role of mGlu2/3 receptors in methamphetamine addiction is peculiar.

Here, we used the novel object recognition (NOR) test to examine whether endogenous activation of either mGlu2 or mGlu3 receptors has any role in the development of cognitive dysfunction in mice repeatedly injected with methamphetamine. The NOR task evaluates the ability to discriminate between a novel and familiar object (Antunes and Biala, 2012) and is a valuable test for the assessment of methamphetamine-induced cognitive dysfunction (Bisagno et al., 2002; Kamei et al., 2006; Reichel et al., 2011). The prefrontal cortex (PFC) and its connected circuits play a key role for recognition memory of objects in the NOR test (Schacter and Slotnick, 2004; Morici et al., 2015; Chao et al., 2022).

We now report that methamphetamine treatment in mice selective enhances mGlu2/3 receptor expression in the PFC, and genetic deletion or selective pharmacological blockade of either mGlu2 or mGlu3 receptors corrects cognitive deficit in the NOR test in methamphetamine-treated mice. In addition, we report that methamphetamine treatment causes an increased expression of proteins related to vesicular glutamate release in the PFC and an inverse operation of both receptors in regulating glutamate release from presynaptic terminals.

## Materials and Methods

### Materials

The following materials were used for the experiments: methamphetamine hydrochloride (Sigma-Aldrich); [^3^H]-D-aspartate (11.3 Ci/mmol, PerkinElmer); myo-inositol [2-^3^H(N)] (15–20 Ci/mmol; Biotrend Chemikalien, CliniSciences, catalog ART-0116A-250); (−)-2-oxa-4-aminocyclo[3.1.0]hexane-4,6-dicarboxylic acid (LY379268, Tocris Bioscience, catalog #2453); VU6001966 (Tocris Bioscience, catalog #6928); (RS)-3,5-dihydroxyphenylglycine (DHPG, Tocris Bioscience, catalog #0342); VU0650786 (MedChemExpress, catalog #HY-108710); lithium chloride (Sigma-Aldrich, catalog #203637); forskolin (FSK, Sigma-Aldrich, catalog #F3917); and 3-isobutyl-1-methylxanthine (IBMX, Sigma-Aldrich, catalog #I5879).

### Animals

mGlu2^−/−^ and mGlu3^−/−^ mice (CD1 background) were originally provided by Eli Lilly and Company and bred at IRCCS Neuromed. Wild-type and knock-out mice were generated by homozygous breadings. Wild-type mice were directly related to the knock-out breading colonies and maintained in parallel. Three-month-old male mice were used for the experiments. We selected male mice to avoid any potential interference of ovarian steroids on behavioral responses to methamphetamine (Eisinger et al., 2018). Mice were housed under controlled conditions (temperature, 22°C; humidity, 40%) with a 12 h light/dark cycle and food and water *ad libitum*. Studies were performed in accordance with the EU Directive 2010/63/EU for animal experiments. The study was approved by the Italian Ministry of Health (Authorization Number 307/2016-PR). All efforts were made to minimize animal suffering and reduce the number of animals used.

### Experimental strategies

All experiments were carried in the light phase of the light/dark cycle. Wild-type, mGlu2^−/−^, and mGlu3^−/−^ mice were intraperitoneally (i.p.) injected with either saline or methamphetamine (1 mg/kg) once a day for 5 d. After 7 d of withdrawal, mice were subjected to the NOR test. Mice were killed after the behavioral test, and dissected brains were used for Western blot analysis in the PFC, NAc, or the hippocampus or immunohistochemical analysis in the perirhinal cortex. Additional groups of wild-type mice treated with either saline or methamphetamine and not exposed to behavioral tests were also used for Western blot analysis of mGlu2/3 receptors.

Separate groups of wild-type, mGlu2^−/−^, and mGlu3^−/−^ male mice treated with saline or methamphetamine were subjected to the open-field test for the assessment of locomotor activity after 7 d of withdrawal.

Finally, individual groups of saline- or methamphetamine-treated wild–type mice were used to examine whether pharmacological blockade of either mGlu2 or mGlu3 receptor could affect behavioral performance in the NOR test. Thirty minutes before the training phase, negative allosteric modulators (NAMs) of mGlu2 (VU6001966, 10 mg/kg) or mGlu3 receptors (VU0650786, 30 mg/kg) were injected intraperitoneally at behaviorally active doses (Engers et al., 2015; Joffe et al., 2020). Control mice were injected intraperitoneally with vehicle (DMSO, 40 µl).

Other groups of saline- or methamphetamine-treated wild–type mGlu2^−/−^ and mGlu3^−/−^ mice were killed after 7 d of withdrawal for ex vivo assessment of mGlu2/3 receptor-mediated inhibition of cAMP formation and mGlu3–mGlu5 receptor cross talk in PFC slices and depolarization-induced [^3^H]-D-aspartate release in superfused PFC synaptosomes.

### NOR test

Mice were subjected to a 3 d protocol test (Golub et al., 2015): (1) habituation (Day 1), mice were individually placed in an open arena (45 × 30 × 30 cm) for 30 min; (2) training (Day 2), 24 h after habituation, mice were placed in the center of the arena containing two identical objects for 15 min; and (3) retention (Day 3), 24 h after training, mice were placed for 3 min in the center of the arena where a novel object replaced one of the familiar objects. The time spent exploring each object during the retention session was recorded and a discrimination index (DI) was calculated as follows: DI = (times spent exploring novel object) / (total time exploring both objects).

### Open-field test

Locomotor activity was monitored in an open-field apparatus using boxes (42 × 42 × 21 cm) in association with an activity monitor equipped with infrared photobeam interruption sensor (Open Field Activity System Hardware, Med Associates). Mice were individually placed into the test box in a quiet room, and locomotor activity was recorded for 60 min with 10 min intervals.

### Measurement of [^3^H]-D-aspartate release in synaptosomal preparation

Synaptosomes were isolated from frozen PFC (Dunkley et al., 1988; Franklin and Taglialatela, 2016; Cisani et al., 2020; Busceti et al., 2021). Tissues were homogenized in 10 volume of 0.32 M sucrose, buffered to pH 7.4 with Tris-(hydroxymethyl)-amino methane (TRIS, final concentration 0.01 M) using a glass/Teflon tissue grinder (clearance 0.25 mm). The homogenate was centrifuged at 1,000 × *g* for 5 min, and the supernatant was gently stratified on a discontinuous Percoll gradient (6, 10, and 20% *v*/*v* in Tris-buffered sucrose) and centrifuged at 33,500 × *g* for 5 min. The layer between 10 and 20% Percoll (synaptosomal fraction) was collected and washed by centrifugation. Synaptosomes were resuspended in a physiological solution (in mM: 140 NaCl; 3 KCl; 1.2 MgSO_4_; 1.2 CaCl_2_; 1.2 NaH_2_PO_4_,; 5 NaHCO_3_; 10 HEPES; 10 glucose), pH 7.2–7.4.

Synaptosomes were incubated for 15 min at 37°C in a rotary water bath in the presence of [^3^H]-D-aspartate (f.c. 50 nM), an unmetabolizable analog of glutamate that mimics efficiently the uptake, the vesicular storage, and the exocytosis of glutamate from synaptosomes (Grilli et al., 2004). Identical portions of synaptosomal suspensions were then layered on microporous filters at the bottom of parallel thermostated chambers in a superfusion system (Raiteri et al., 1974; Ugo Basile) and superfused at a flow rate of 0.50 ml/min. Synaptosomes were superfused for 39 min and then transiently (90 s) exposed, at *t* = 39 min, to 15 mM KCl containing medium (NaCl substituting for an equimolar concentration of KCl), then replaced with standard medium until the end of the superfusion (*t *= 48 min). When indicated, LY379268 (1–10 nM) was added concomitantly to the depolarizing stimulus as previously described (Taddeucci et al., 2022).

Fractions were collected as follows: two 3 min fractions (basal release), one before (*t* = 36–39 min) and one after (*t* = 45–48 min) a 6 min fraction (*t* = 39–45 min) containing the evoked release. Fractions collected and superfused synaptosomes were measured for radioactivity. The amount of radioactivity released into each superfusate fraction was expressed as the percentage of the total radioactivity. The 15 mM KCl-evoked [^3^H]-D-aspartate overflow was estimated by subtracting the neurotransmitter content into the first and the third fractions collected (basal release) from that in the 6 min fraction collected during and after the depolarization pulse (evoked release), and it is expressed as induced overflow over basal release (%).

### Measurement of cAMP formation and PI hydrolysis in PFC slices

For measurements of cAMP formation, PFC coronal slices (1 mm thick) were cut with a brain matrix and incubated in a Krebs–Henseleit buffer (equilibrated with 95% O_2_/5% CO_2_), pH 7.4, at 37°C in a shaker for 45 min under constant oxygenation. Slices were preincubated with the phosphodiesterase inhibitor IBMX (0.5 mM) and treated 15 min after with LY379268 (3 µM) or vehicle followed, 2 min later, by the adenylyl cyclase stimulator FSK (10 µM) or its vehicle. After 20 min of incubation, the reaction was stopped by adding ice-cold 0.4 N perchloric acid. Samples were sonicated for 10–15 s, added to 2 NK_2_CO_3_, and centrifuged at 600 × *g*. cAMP levels were measured in the supernatant by using an enzyme-linked immunoassay kit (TEMA Ricerca). PI hydrolysis was measured as reported previously (Di Menna et al., 2018). In brief, 350 × 350 µm PFC slices were incubated in a Krebs–Henseleit buffer, pH 7.4, containing 1 µCi myo-inositol [2-^3^H(N)]. After addition of 10 mM LiCl, slices were challenged with 200 µM DHPG alone or combined with LY379268 (3 µM). [^3^H]Inositolmonophosphate (InsP) accumulated in the presence of Li^+^ was separated by anion exchange chromatography. For protein measurements, samples were dried after removal of the water phase and incubated with 0.5 N NaOH at 50°C for 2 h. Proteins were measured as described by Lowry et al. (1951).

### Western blot analysis

Dissection of PFC, hippocampus, and NAc were homogenized at 4°C in an ice-cold lysis buffer. Western blot analysis was performed loading the following quantity of proteins in a sample buffer containing 10% 2-mercaptoethanol: (1) 2.5 µg for Rab3A (Ras-related protein 3A), Munc-18 (mammalian homolog of the unc-18 gene), and Syn (synaptophysin); (2) 5 µg for STX 1A (syntaxin 1A) and xCT (cystine-glutamate antiporter); or (3) 10 µg for mGlu2/3, vGlut1 (vesicular glutamate transporter 1), and AGS3 (Type 3 activator of G-protein signaling). Proteins were resuspended in sodium dodecylsulfate (SDS)-bromophenol blue reducing buffer and separated with SDS polyacrylamide gels (8% for mGlu2,3, vGlut1, and AGS3; 12% for Rab3A, Munc-18, STX 1A, Syn, and xCT).

Gels were electroblotted on polyvinyldene fluoride membranes and filters blocked in 5% nonfat dry milk [60 min at room temperature (RT)] and then overnight incubated at 4°C with the following rabbit polyclonal (*rp*) or mouse monoclonal (*mm*) primary antibodies: *rp* anti-mGlu2/3 (Sigma-Aldrich; catalog #G9790; RRID, AB_259998; 1:500); *rp* anti-vGlut1 (Cell Signaling Technology; catalog #12331; RRID, AB_2797887; 1:500); *rp* anti-Rab3A (Thermo Fisher Scientific; catalog #PA1-4691; RRID, AB_2177399; 1:25,000); *rp* anti-Syn (Cell Signaling Technology, catalog #5461 s, 1:25,000); *rp* anti-Munc-18 (Abcam; catalog #ab124920; RRID, AB_10976239; 1:5,000,000); *mm* anti-xCT (TransGenic, catalog #KE021, 1:2,000); *mm* anti-AGS3 (Santa Cruz Biotechnology; catalog #sc-136482; RRID, AB_10608965; 1:300); *mm* anti-STX 1A (Merck Millipore, catalog #MAB336-C, 1:10,000); *mm* anti-glyceraldehyde-3-phosphate dehydrogenase (Santa Cruz Biotechnology; catalog #sc-32233; RRID, AB_627679; 1:1,000), or *mm* anti-β-actin (Santa Cruz Biotechnology; catalog #sc69879; RRID, AB_1119529; 1:1,000). Afterward, filters were incubated for 60 min at RT with the following secondary peroxidase conjugated antibodies: anti-rabbit IgG (Merck Millipore, catalog #401393, 1:3,000) or anti-mouse IgG (Immunological Sciences, catalog #IS20400, 1:5,000). Immunostaining was revealed by enhanced chemiluminescence luminosity.

### Immunohistochemical analysis of mGlu2/3 receptors in the perirhinal cortex

Seven days after the last injection of saline or methamphetamine, mice were killed, and brains were fixed in Carnoy's solution (60% ethanol, 10% acetic acid, 30% chloroform) and embedded in paraffin. Ten micrometer of serial sections of the perirhinal cortex were cut with a microtome (Leica RM2245) and used for immunohistochemical analysis of mGlu2/3 receptors. Deparaffinized sections were treated with formic acid (70%, 30 min at RT) for antigen retrieval. Afterward, sections were permeabilized in Triton X-100 and then incubated with phosphate buffer solution (PBS)/6% normal horse serum. Slices were then incubated overnight at 4°C with a rabbit polyclonal anti-mGlu2/3 receptor antibody (1:50; Sigma-Aldrich; catalog #G9790; RRID, AB_259998). Finally, slices were incubated for 60 min at RT with a secondary biotinylated anti-rabbit IgG made in horse (Vector Laboratories; code, BA1100; 1:200; RRID, AB_2336201) and then for 60 min at RT with Alexa Fluor 568 conjugated streptavidin (Life Technologies; code, S11226; 1:200). Negative controls were performed on separate sections stained in the absence of primary antibodies. The staining was analyzed by a fluorescence microscope. Images of the regions of interest were digitally collected and counted by the ImageJ software.

### Statistical analysis

**Figure 1.**
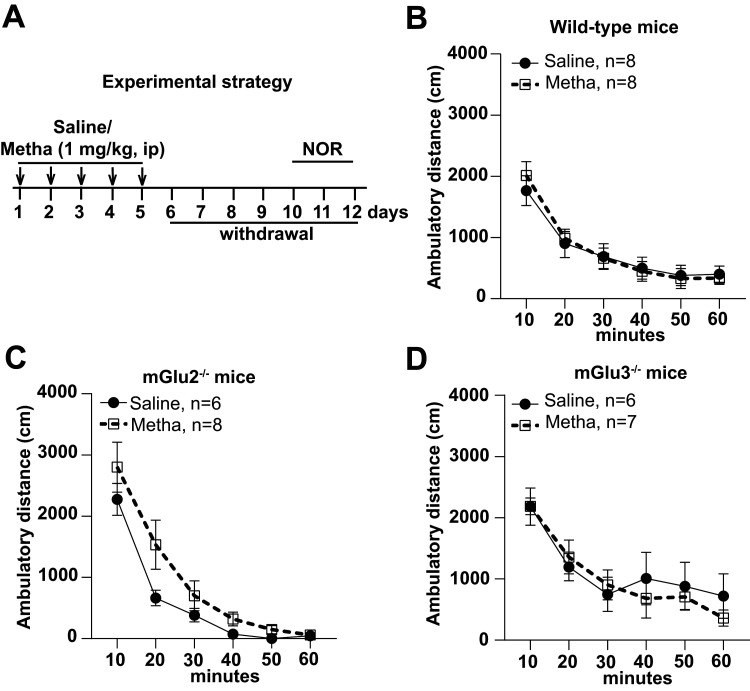
Methamphetamine treatment does not affect motor activity at Day 7 of withdrawal. The protocol used for behavioral assessment of methamphetamine-induced cognitive dysfunction in the NOR test is shown in ***A***. Locomotor activity in the open-field apparatus at Day 11 (6 d of withdrawal) is shown for wild-type (***B***), mGlu2^−/−^ (***C***), and mGlu3^−/−^ (***D***) mice treated with saline or methamphetamine. Data are means ± SEM.

**Figure 2.**
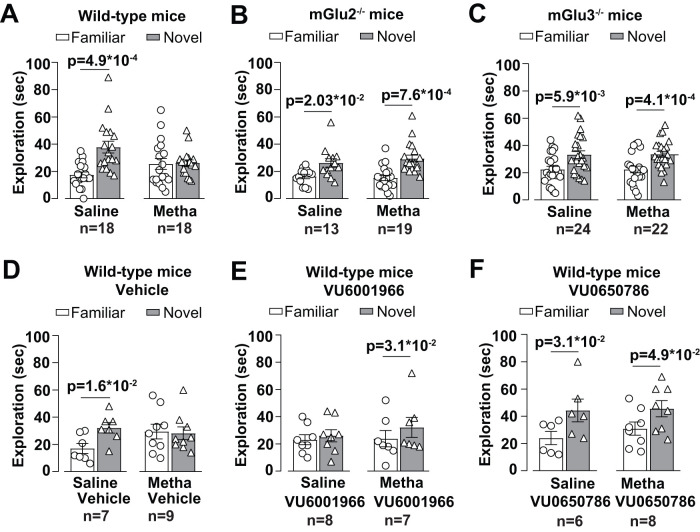
mGlu2/3 receptors genetic deletion or pharmacological blockade abrogates methamphetamine (Metha)-induced memory deficit in mice. Exploration time in seconds (sec) during the retention phase in the NOR task in wild-type (***A***), mGlu2^−/−^ (***B***), and mGlu3^−/−^ (***C***) mice treated with saline or Metha (1 mg/kg, i.p.) for 5 consecutive days. All mice were tested after 7 d of withdrawal (i.e., day 9 d 11). ***D–F***, The performance on the NOR task for wild-type mice after 7 d of withdrawal following the same treatment with saline or Metha and injected 30 min before the training phase in the NOR paradigm with vehicle (DMSO, 40 µl i.p.), the selective negative modulator of the mGlu2 (VU6001966, 10 mg/kg, i.p.), or mGlu3 (VU0650786, 30 mg/kg, i.p.) receptor, respectively. Data are means ± SEM: Wilcoxon two-tailed matched-pair signed–rank test in ***A***, ***B***, ***D***, and ***E*** (metha) and ***F*** (saline); two-way ANOVA for repeated measures with Bonferroni’s for multiple comparisons (novel vs familiar, *F*_(1,44) _= 23.72; *p* = 1.5  *10^−5^; treatment, *F*_(1,44) _= 0.0021; *p* = 0.96; interaction, *F*_(1,44) _= 0.0059; *p* = 0.94) in ***C***; paired two-tailed Student’s *t* test in ***F***, metha (*t* = 2.375; df = 7). *p values are shown in the figure.

**Figure 3.**
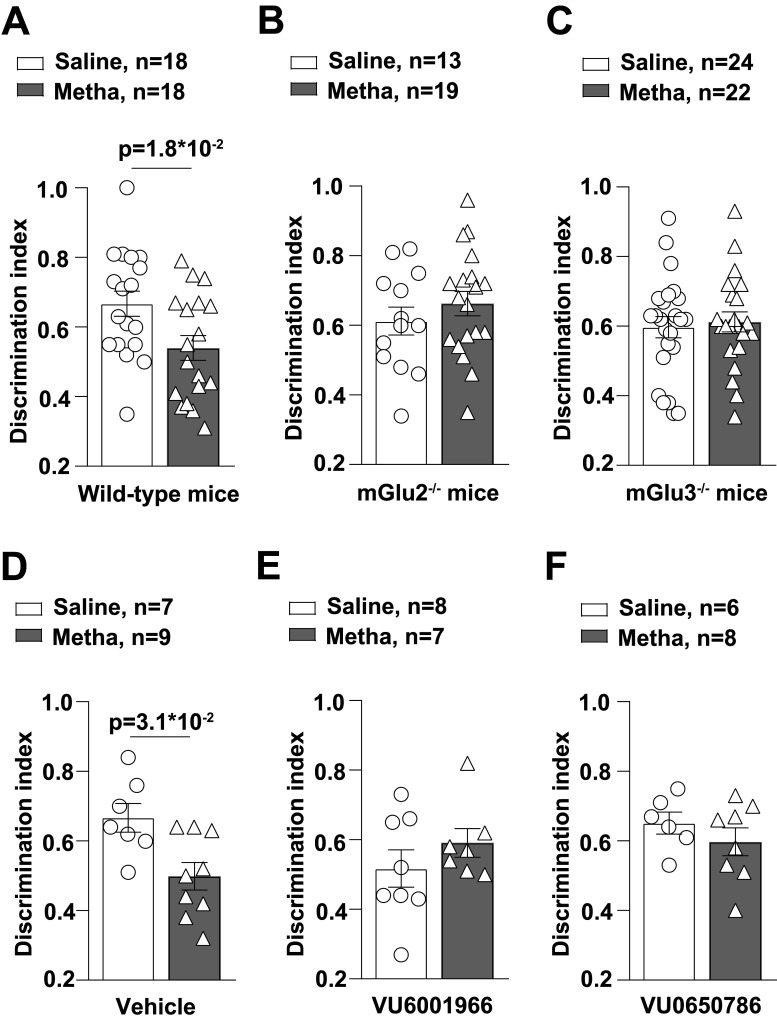
DI for wild-type, mGlu2^−/−^, and mGlu3^−/−^ mice subjected to the NOR test following treatment with saline or methamphetamine (Metha). DI was calculated as a measure of relative novelty preference as follows: DI = (times spent exploring novel object) / (total time exploring both objects). ***A*–*C***, Values of DI for wild-type, mGlu2^−/−^, and mGlu3^−/−^ mice subjected to the NOR test after 7 d of withdrawal following treatment with saline or Metha. Values of DI for wild-type mice treated with saline or Metha (1 mg/kg, i.p. for 5 consecutive days) and subjected to the NOR test after 7 d of withdrawal in the presence or absence of NAMs of mGlu2 (VU6001966) or mGlu3 (VU0650786) receptors are shown in ***D–F***, respectively. Vehicle (DMSO, 40 µl i.p.) or the respective compounds (VU6001966, 10 mg/kg, i.p.; VU0650786, 30 mg/kg, i.p.) were injected 30 min before the training phase in the NOR paradigm. Data are means ± SEM: unpaired *t* test in ***A*** (*t* = 2.485, df = 34); Mann–Whitney in ***D*** (*U* = 11). *p values are shown in the figure.

**Figure 4.**
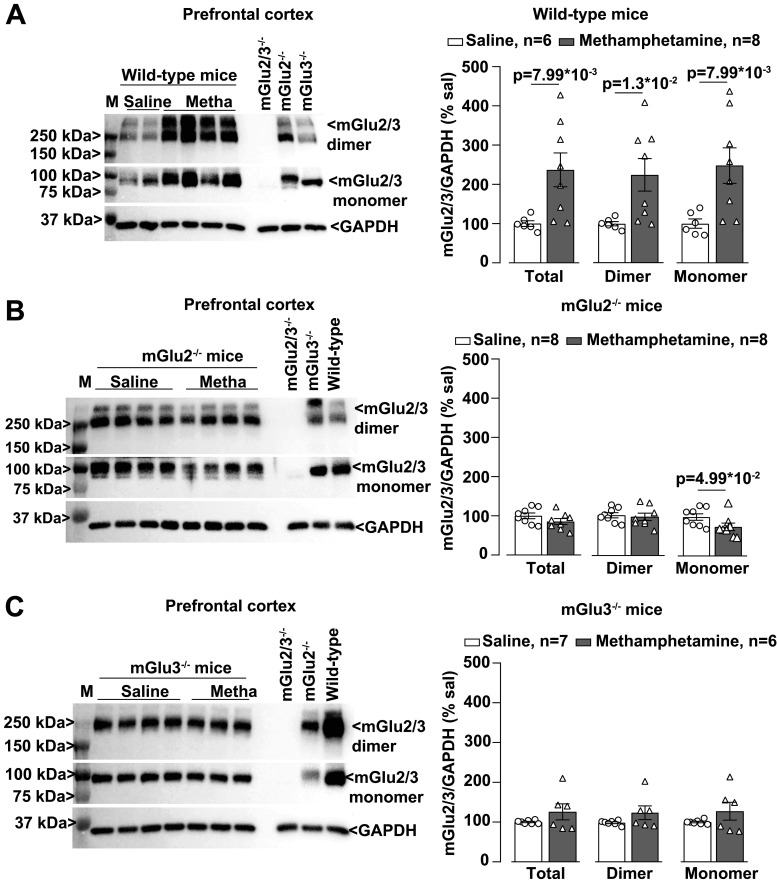
mGlu2/3 expression is upregulated in the PFC of methamphetamine (Metha)-treated wild–type mice. Western blot analysis for mGlu2/3 receptors expression in the PFC of wild-type (***A***), mGlu2^−/−^ (***B***), and mGlu3^−/−^ (***C***) mice treated with saline or Metha (1 mg/kg, i.p.) for 5 consecutive days. All mice were killed after 7 d of withdrawal (i.e., Day 11). Prestained protein standards were loaded on the first lane as molecular mass markers (M). Densitometric values of the dimer (250 kDa), the monomer (100 kDa), or the sum of dimer + monomer (total) were analyzed. Protein extracts from the PFC of wild-type mice or mGlu2^−/−^, mGlu3^−/−^, or mGlu2,3^−/−^ mice were used as control/negative controls. Data are means ± SEM: Mann–Whitney two-tailed test [*U* = 4 for ***A*** (total) and ***A*** (dimer); *U* = 5 for ***A*** (monomer); *U* = 13 for ***B*** (monomer)]. *p values are shown in the figure.

Statistical analysis was performed using GraphPad Prism (GraphPad Software, version 8.0.1). Normal distribution was assessed by the D'Agostino and Pearson’s test. For data showing a normal distribution, we used the following parametric statistical analysis: paired two-tailed Student’s *t* test (Fig. 2*E*, saline; *F*, methamphetamine) or unpaired two-tailed Student’s *t* test (Figs. 3*A*–*C*, 4*B*, total; 4*B*, dimer; 5*A*; 6*A–C*, total; 6*C*, dimer; 7; 9*A′*; 11*A*, mGlu2^−/−^ mice; 11*C*, mGlu2^−/−^ mice; 12*D*, mGlu2^−/−^ mice) for two-group comparison and one-way ANOVA (Figs. 9*A*; 9*D*, saline; 10*A*, saline; 10*B*, saline; 10*C*, methamphetamine) or Two-way ANOVA for repeated measures (Fig. 2*C*) with the Bonferroni’s test for multiple comparison. When data did not follow a normal distribution or for *n* ≤ 6 per group, data were analyzed with the Mann–Whitney two-tailed test (Figs. 1, 3*D*–*F*, 4*A*,*B*, monomer; 4*C*; 5*B,C*; 6*C*, monomer; 8; 9*B′–D′*; 11*A*, wild-type mice; 11*A*, mGlu3^−/−^ mice; 11*B*,*C*, wild-type mice; 11*C*, mGlu3^−/−^ mice; 12*A*–*D*, wild-type mice; 12*D*, mGlu3^−/−^ mice) and Wilcoxon two-tailed test (Fig. 2*A,B*,*D*,*E*, methamphetamine; *F*, saline) for two-group comparison or the Kruskal–Wallis test (Fig. 9*B–D*, methamphetamine; 10*A*, methamphetamine; 10*B*, methamphetamine; 10*C*, saline) followed by Dunn's test for comparison across multiple groups. *P* values <0.05 were considered statistically significant. The precise *p* values and sample numbers (*n*) were stated in the figures. Details on error bars and statistical analysis can be found in figure legends.

## Results

Mice treated daily for 5 d with either saline or methamphetamine (1 mg/kg) were trained in the NOR test 7 d after the last injection (Fig. 1*A*). This protocol was necessary to avoid the effect of methamphetamine on motor activity (Fig. 1*B–D*). Wild-type mice treated with saline showed the expected performance in the NOR test, spending more time in exploring the novel object. Recognition of the novel object was abolished after treatment with methamphetamine (Figs. 2*A*, 3*A*). In contrast, both mGlu2^−/−^ and mGlu3^−/−^ mice were resilient to methamphetamine-induced cognitive dysfunction in the NOR test (Figs. 2*B*,*C*, 3*B*,*C*). To strengthen these findings, we used a pharmacological approach using selective mGlu2 or mGlu3 receptor NAMs (compounds VU6001966 and VU0650786, respectively). VU6001966 and VU0650786 were injected intraperitoneally at doses of 10 and 30 mg/kg, respectively (Engers et al., 2015; Joffe et al., 2020). Mice were treated with the two compounds or their vehicle 30 min before training. Both treatments abolished methamphetamine-induced cognitive impairment in the NOR test (Figs. 2*D*–*F*; 3*D*–*F*).

**Figure 5.**
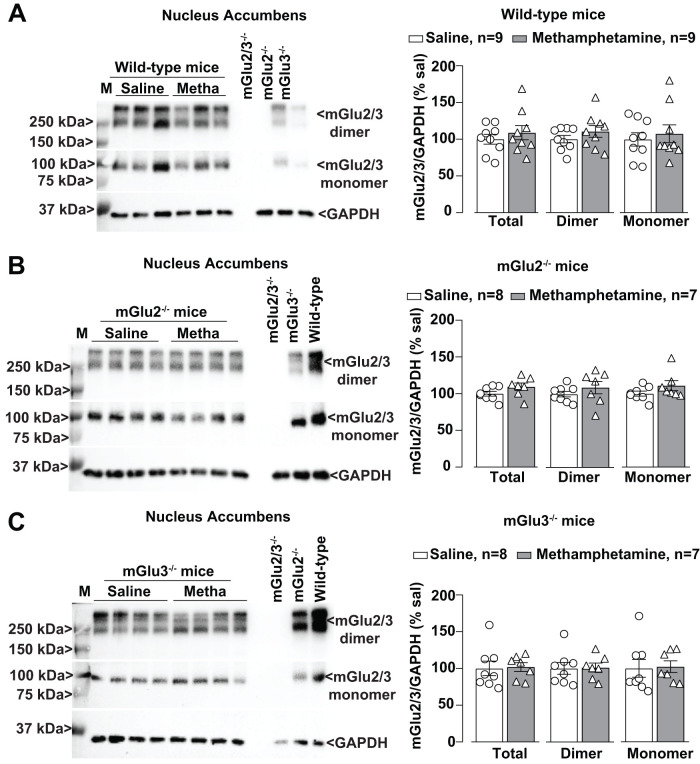
Methamphetamine (Metha) treatment does not affect the mGlu2/3 receptor expression in the NAc of wild-type, mGlu2^−/−^, and mGlu3^−/−^ mice. Western blot analysis for mGlu2/3 receptors expression in the NAc of wild-type (***A***), mGlu2^−/−^ (***B***), and mGlu3^−/−^ (***C***) mice treated with saline or Metha (1 mg/kg, i.p.) for 5 consecutive days. Mice were subjected to the NOR test at 7 d of withdrawal following the treatment with saline or Metha. All mice were killed after the NOR test. Prestained protein standards were loaded on the first lane as molecular mass markers (M). Densitometric values of the dimer (250 kDa), the monomer (100 kDa), or the sum of dimer + monomer (total) were analyzed. Protein extracts from the NAc of wild-type mice or mGlu2^−/−^, mGlu3^−/−^, or mGlu2,3^−/−^ mice were used as control/negative controls. Data are means ± SEM.

**Figure 6.**
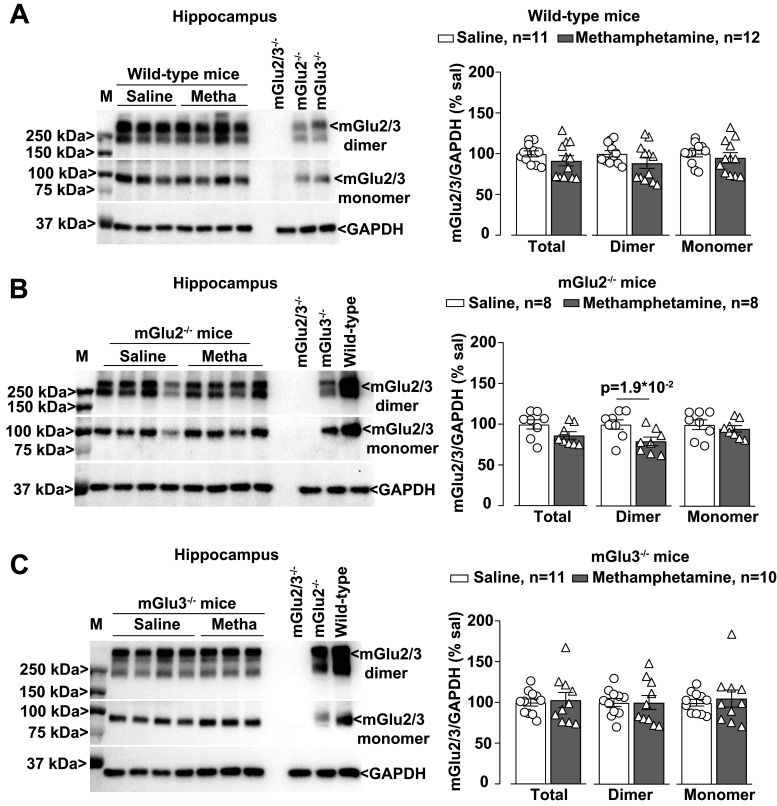
Methamphetamine (Metha) treatment does not affect the mGlu2/3 receptor expression in the hippocampus of wild-type, mGlu2^−/−^, and mGlu3^−/−^ mice. Western blot analysis for mGlu2/3 receptors expression in the hippocampus of wild-type (***A***), mGlu2^−/−^ (***B***), and mGlu3^−/−^ (***C***) mice treated with saline or Metha (1 mg/kg, i.p.) for 5 consecutive days. Mice were subjected to the NOR test at 7 d of withdrawal following the treatment with saline or Metha. All mice were killed after the NOR test. Prestained protein standards were loaded on the first lane as molecular mass markers (M). Densitometric values of the dimer (250 kDa), the monomer (100 kDa), or the sum of dimer + monomer (total) were analyzed. Protein extracts from the hippocampus of wild-type mice or mGlu2^−/−^, mGlu3^−/−^, or mGlu2,3^−/−^ mice were used as control/negative controls. Data are means ± SEM.

**Figure 7.**
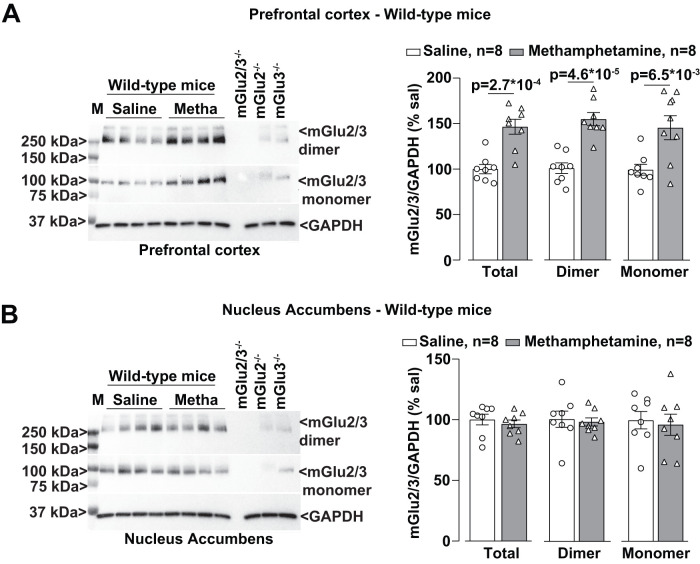
Exposure to NOR does not affect the mGlu2/3 receptor expression in the PFC and NAc of wild-type mice. Western blot analysis for mGlu2/3 receptors expression in the PFC (***A***) and NAc (***B***) of wild-type mice treated with saline or methamphetamine (Metha; 1 mg/kg, i.p.) for 5 consecutive days. All mice were killed after 7 d of withdrawal (i.e., Day 11). Mice were not subjected to the NOR test. Prestained protein standards were loaded on the first lane as molecular mass markers (M). Densitometric values of the dimer (250 kDa), the monomer (100 kDa), or the sum of dimer + monomer (total) were analyzed. Protein extracts from the PFC (***A***) or NAc (***B***) of mGlu2^−/−^, mGlu3^−/−^, or mGlu2,3^−/−^ mice were used as negative controls. Data are means ± SEM. Unpaired two-tailed Student’s *t* test *t* (*t* = 4.821; df = 14 for PFC dimer + monomer; *t* = 3.191; df = 14 for PFC monomer; *t* = 5.796; df = 14 for PFC dimer). *p values are shown in the figure.

We then examined whether methamphetamine treatment could induce changes in the expression of mGlu2/3 receptors in the PFC, NAc, and hippocampus. Western blot analysis with an antibody anti-mGlu2/3 showed two partially overlapping bands, which correspond to mGlu2 and mGlu3 receptor monomers (see lanes loaded with the tissue from mGlu2^−/−^ or mGlu3^−/−^ mice in Fig. 4*A*), and higher-molecular-size bands that might correspond to receptor dimers (Fig. 4*A*). Methamphetamine treatment caused a large upregulation of mGlu2/3 receptors in the PFC (Fig. 4*A*), but not in the NAc or hippocampus, of wild-type mice (Figs. 5*A*, 6*A*). No changes were seen in the PFC of mGlu2^−/−^ or mGlu3^−/−^ mice (Fig. 4*B,C*), suggesting that methamphetamine enhanced the expression of mGlu2/3 receptor heterodimers in the PFC. Methamphetamine did not cause changes in mGlu2/3 protein levels in the NAc or hippocampus of mGlu2^−/−^ or mGlu3^−/−^ mice (Figs. 5*B*,*C*, 6*B*,*C*). mGlu2/3 receptor protein levels were also increased in the PFC of methamphetamine-treated wild–type mice which had not been trained in the NOR test (Fig. 7*A*,*B*).

**Figure 8.**
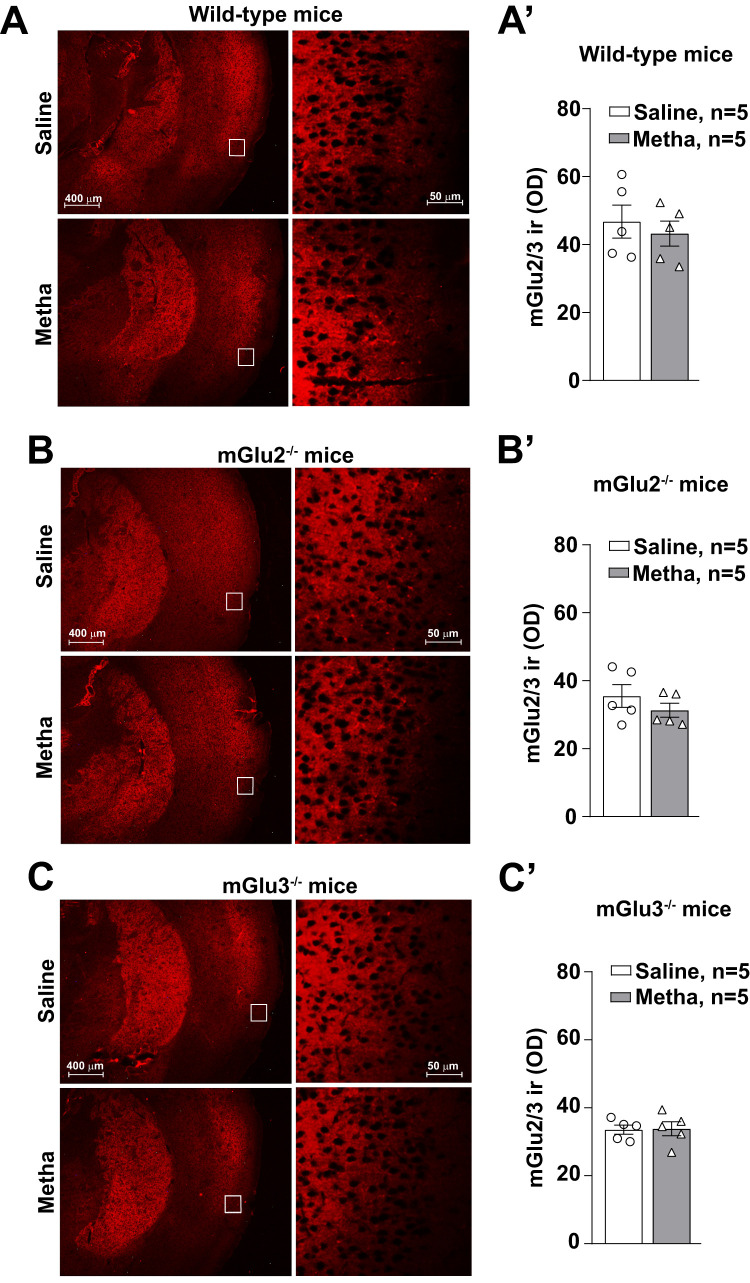
Methamphetamine (Metha) treatment does not affect the mGlu2/3 receptor expression in the perihinal cortex of wild-type, mGlu2^−/−^, and mGlu3^−/−^ mice. Immunohistochemical analysis for mGlu2/3 receptors expression in the perirhinal cortex of wild-type (***A***), mGlu2^−/−^ (***B***), and mGlu3^−/−^ (***C***) mice treated with saline or Metha (1 mg/kg, i.p.) for 5 consecutive days. Mice were subjected to the NOR test at 7 d of withdrawal following the treatment with saline or Metha. All mice were killed after the NOR test. Densitometric values of mGlu2/3 immunoreactivity (ir) in the perirhinal cortex of wild-type, mGlu2^−/−^, and mGlu3^−/−^ mice are shown in ***A*′***–**C*****′**, respectively.

We also examined mGlu2/3 receptor expression in the perirhinal cortex, which contributes to NOR memory (Barker et al., 2007). The analysis was performed by immunohistochemistry for technical reasons. mGlu2/3 receptor density was unchanged in response to methamphetamine in the three genotypes (Fig. 8).

**Figure 9.**
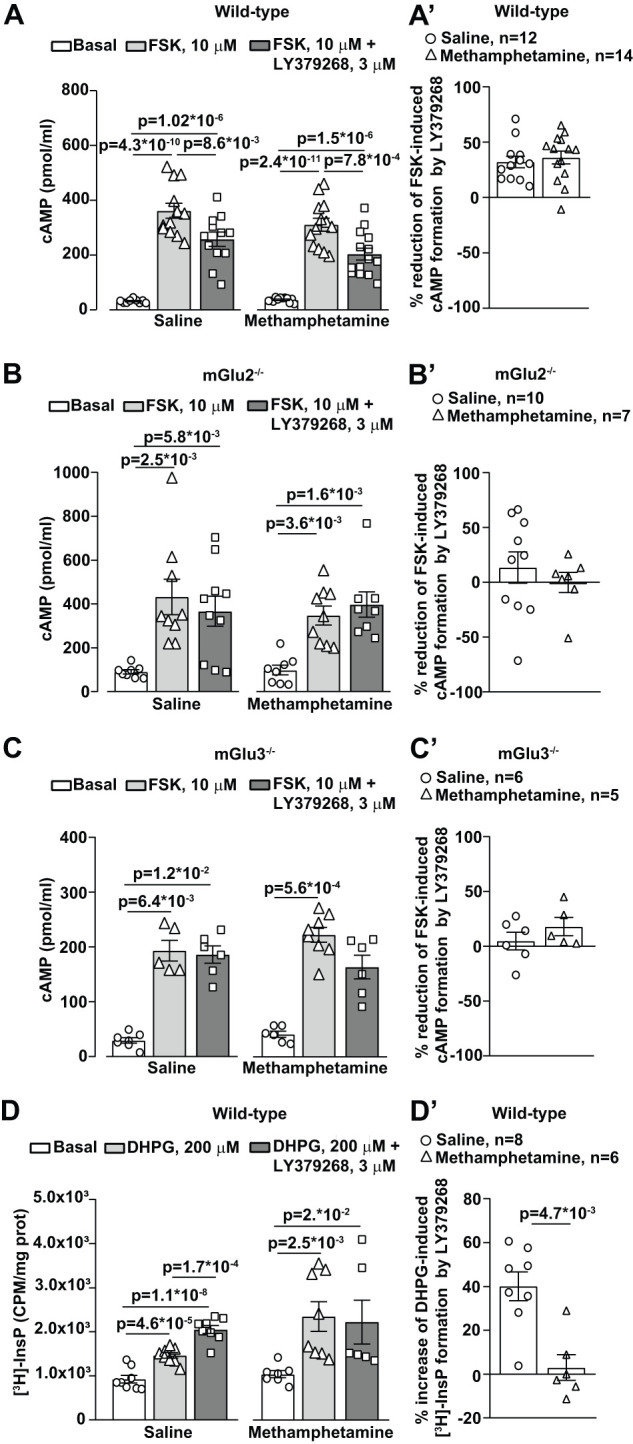
Effect of methamphetamine treatment on signal transduction mechanisms of mGlu2 and mGlu3 receptors in the PFC. cAMP levels in PFC slices from wild-type, mGlu2^−/−^, and mGlu3^−/−^ mice treated with saline or methamphetamine (1 mg/kg, i.p.), for 5 consecutive days and killed after 7 d of withdrawal are shown in ***A–C***, respectively. Slices were pretreated with LY379268 (3 µM, 10 min before FSK) and then stimulated for 20 min with FSK (10 µM). Graphs in ***A*′*–C*′** show the percentage of reduction of FSK-induced cAMP formation by LY379268 for each genotype. Values are means ± SEM: (***A***) One-way ANOVA with Bonferroni's multiple-comparison test (*F*_(2,30) _= 46.11; *p* = 7.1  *10^−10^ for saline; *F*_(2,36) _= 49.54; *p* = 4.6  *10^−11^ for methamphetamine); (***B,C***) Kruskal–Wallis test with Dunn's multiple-comparison test (Kruskal–Wallis statistic, 13.55 in ***B***, saline; 14.9 in ***B***, methamphetamine; 12.29 in ***C***, saline; 14.01 in ***C***, methamphetamine). *p values are shown in the figure. ***D***, Stimulation of PI hydrolysis by DHPG combined or not with LY379268 in slices of PFC prepared from wild-type mice treated with saline or methamphetamine (1 mg/kg, i.p.), for 5 consecutive days and killed after 7 d of withdrawal. Slices were treated with DHPG (200 µM, 60 min) in the absence or presence of LY379268 (3 µM). LY379268 was not tested in the absence of DHPG because it is known to be ineffective on PI hydrolysis on its own in the adult mouse brain (Di Menna et al., 2018). Values are means ± SEM: saline, One-way ANOVA with Bonferroni's multiple-comparison test (*F*_(2,21) _= 46.22; *p* = 2.03  *10^−8^); methamphetamine, Kruskal–Wallis test with Dunn's multiple-comparison test (Kruskal–Wallis statistic, 12.5). *p values are shown in the figure.

We then examined the primary signal transduction mechanism of mGlu2/3 receptors, i.e., the inhibition of adenylyl cyclase activity, in PFC slices. Inhibition of FSK-stimulated cAMP formation by the mGlu2/3 receptor agonist, LY379268, was unchanged in slices prepared from wild-type mice treated with methamphetamine (Fig. 9*A*) and was blunted in slices prepared from either mGlu2^−/−^ or mGlu3^−/−^ mice (Fig. 9*B*,*C*). Knowing that postsynaptic mGlu3 receptors are functionally linked to mGlu5 receptors and boost mGlu5 receptor-mediated PI hydrolysis (Di Menna et al., 2018), we measured the PI response to the mGlu1/5 receptor agonist, DHPG (200 µM) combined or not with LY379268 (3 µM), in PFC slices. In slices from control mice, LY379268 amplified DHPG-stimulated [^3^H]InsP formation hydrolysis, as expected. In slices prepared from methamphetamine-treated mice, DHPG increased [^3^H]InsP formation to a greater extent, but the DHPG response was unaffected by LY379268 (Fig. 9*D*).

**Figure 10.**
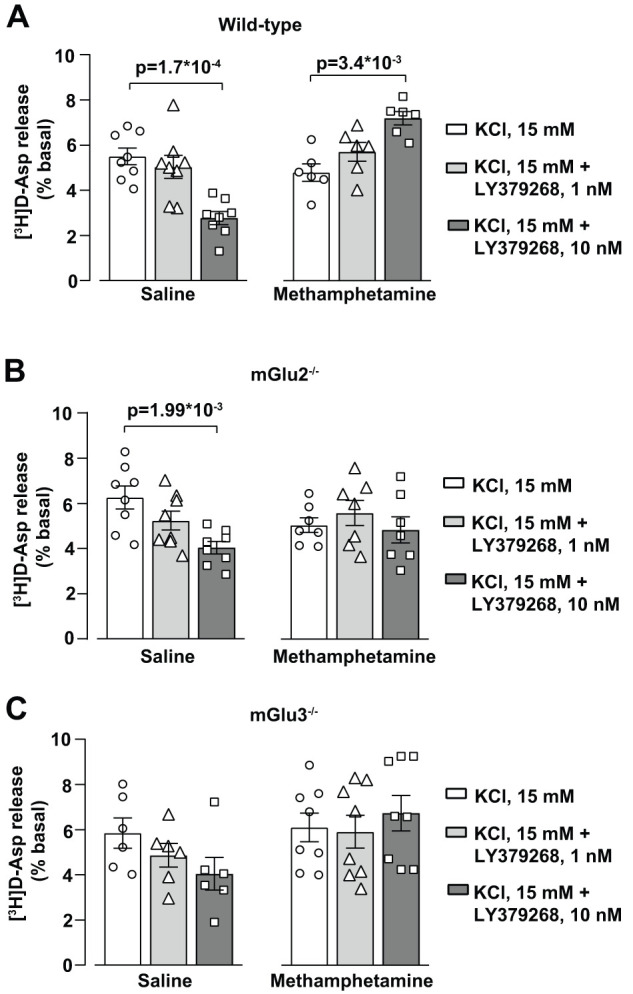
Methamphetamine treatment alters the modulation of [^3^H]-D-aspartate release by mGlu2/3 receptors in PFC synaptosomes. Depolarization-induced [^3^H]-D-aspartate (Asp) release in superfused synaptosomes prepared from the PFC of wild-type (***A***), mGlu2^−/−^ (***B***), and mGlu3^−/−^ (***C***) mice treated with saline or methamphetamine (1 mg/kg, i.p.), for 5 consecutive days and killed after 7 d of withdrawal. Synaptosomes were transiently (90 s) exposed to 15 mM KCl containing medium. When indicated, LY379268 (1–10 nM) was added concomitantly to the depolarizing stimulus. Fractions were collected as follows: two 3 min fractions (basal release), one before (*t* = 36–39 min) and one after (*t* = 45–48 min) a 6 min fraction (*t* = 39–45 min) containing the evoked release. The KCl-evoked [^3^H]-D-aspartate overflow was estimated by subtracting the neurotransmitter content into the first and the third fractions collected (basal release) from that in the 6 min fraction collected during and after the depolarization pulse (evoked release), and it is expressed as induced overflow over basal release (%). Values are means + SEM: one-way ANOVA with Bonferroni's multiple-comparison test for ***A***, saline (*F*_(2,21) _= 13.41; *p* = 1.8  *10^−4^), and ***B***, saline (*F*_(2,21) _= 7.31; *p* = 3.9 1.8  *10^−3^); Kruskal–Wallis test with Dunn's multiple-comparison test for ***A***, methamphetamine (Kruskal–Wallis statistic, 10.15). *p values are shown in the figure.

One of the most established function of mGlu2 and mGlu3 receptors is the inhibition of glutamate release from presynaptic terminals (Nicoletti et al., 2011). Superfused synaptosomal preparation represent a clean model to study the regulation of glutamate release by presynaptic receptors because superfusion avoids confounding factors, such as autocrine or paracrine mechanisms affecting glutamate release (Raiteri et al., 1974). Previous studies have shown that the mGlu2/3 receptor agonist, LY379268, showed a high potency in reducing depolarization-evoked glutamate release, being active at concentrations of 10 nM (Cisani et al., 2020), which approximate the reported EC_50_ value of the compound at mGlu2 or mGlu3 receptors (Schoepp et al., 1999). In synaptosomes prepared from the PFC of wild-type mice treated with saline, 10 nM LY379268 halved the stimulation of [^3^H]-D-aspartate release evoked by depolarizing concentrations of K^+^ (15 mM; Fig. 10*A*). The inhibitory action of LY379268 was reduced in synaptosomes prepared from the PFC of either mGlu2^−/−^ or mGlu3^−/−^ mice (Fig. 10*B*,*C*). Surprisingly, LY379268 significantly enhanced depolarization-evoked [^3^H]-D-aspartate release in PFC synaptosomes prepared from wild-type mice treated with methamphetamine (Fig. 10*A*), and this effect was not visible in synaptosomes from mGlu2^−/−^ or mGlu3^−/−^ mice (Fig. 10*B*,*C*).

**Figure 11.**
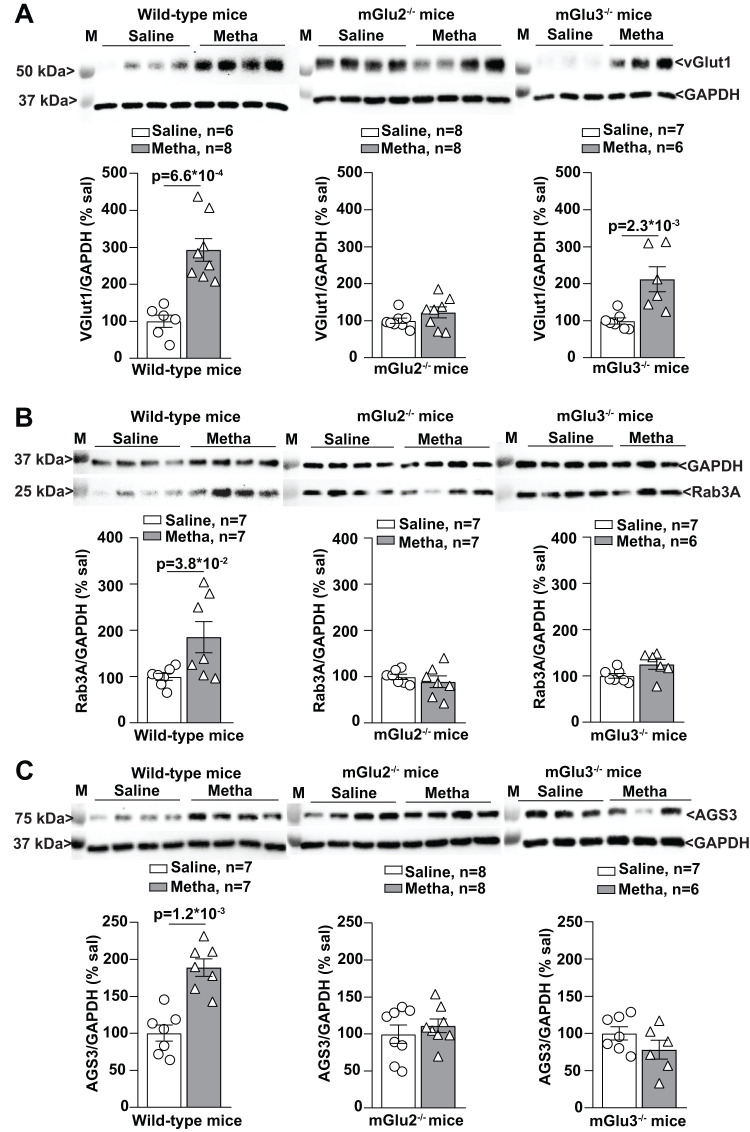
vGlut1 (vesicular glutamate transporter 1), Rab3A (Ras-related protein 3A), and AGS3 (activator of G-protein signaling 3) expression is upregulated in the PFC of methamphetamine (Metha)-treated wild-type mice. Western blot analysis for vGlut1 (***A***), Rab3A (***B***), and AGS3 (***C***) expression in the PFC of wild-type, mGlu2^−/−^, and mGlu3^−/−^ mice treated with saline or Metha (1 mg/kg, i.p.) for 5 consecutive days. All mice were killed after 7 d of withdrawal (i.e., Day 11). Data are means ± SEM: Mann–Whitney two-tailed test (*U* = 0 for ***A*** wild-type mice; *U* = 1 for ***A*** mGlu3^−/−^ mice, and ***C*** wild-type mice; *U* = 8 for ***B*** wild-type mice). *p values are shown in the figure.

**Figure 12.**
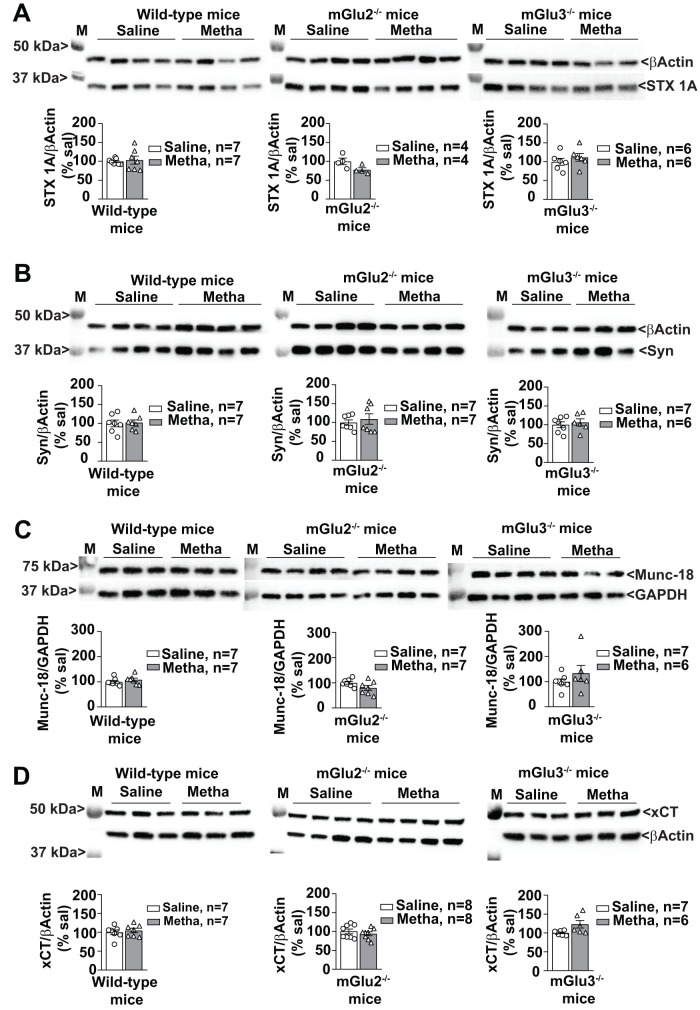
Methamphetamine (Metha) treatment does not affect the expression level of STX 1A (syntaxin 1A), Syn (synaptophysin), Munc-18 (mammalian homolog of the unc-18 gene), and xCT (cystine-glutamate antiporter) in the PFC of Metha-treated of wild-type, mGlu2^−/−^, and mGlu3^−/−^ mice. Western blot analysis for STX 1A (***A***), Syn (***B***), Munc-18 (***C***), and xCT (***D***) expression in the PFC of wild-type, mGlu2^−/−^, and mGlu3^−/−^ mice treated with saline or Metha (1 mg/kg, i.p.) for 5 consecutive days. All mice were killed after 7 d of withdrawal (i.e., Day 11). Prestained protein standards were loaded on the first lane as molecular mass markers (M). Data are means ± SEM.

This unexpected finding encouraged the study of proteins that are specifically related to vesicular release of glutamate such as the vesicular glutamate transporter, vGlut1, Rab3A, and Munc-18. Two proteins nonspecifically associated with neurotransmitter release, e.g., STX 1A and Syn, were also measured. Methamphetamine treatment caused a large increase in vGlut1 protein levels in the PFC of wild-type mice. This effect was abolished in mGlu2^−/−^ mice but was still present, albeit to a lesser extent, in mGlu3^−/−^ mice (Fig. 11*A*). Methamphetamine also caused a significant increase in Rab3A protein levels in the PFC of wild-type mice, but not in the PFC of mGlu2^−/−^ or mGlu3^−/−^ mice (Fig. 11*B*). Levels of STX 1A, Syn, or Munc-18 were unchanged in response to methamphetamine (Fig. 12*A*–*C*).

Finally, we measured proteins that are linked to the function of presynaptic mGlu2/3 receptors, i.e., xCT and AGS3. Presynaptic mGlu2 and mGlu3 receptors are localized in the preterminal region of axon terminals and are not easily accessible to synaptic glutamate (Moussawi and Kalivas, 2010; Bernabucci et al., 2012). It has been suggested that the glutamate released from astrocytes through the xCT antiporter activates presynaptic mGlu2 and mGlu3 receptors (Kalivas, 2009). Methamphetamine treatment failed to induce changes in the levels of xCT (the catalytic subunits of xC^−^) in the PFC of the three genotypes (Fig. 12*D*). In contrast, methamphetamine caused a large increase in the levels of the activator of G-protein signaling, AGS3, in the PFC of wild-type, but not mGlu2^−/−^ or mGlu3^−/−^, mice (Fig. 11*C*). This is an interesting finding because, in spite of its name, AGS3 causes a functional switch in G_i/o_ protein function, driving G-protein signaling toward pathways activated by βɣ rather than α_i/o_ subunits (Lanier, 2004).

## Discussion

Regulation of excitatory synaptic transmission by mGlu2 and mGlu3 receptors is complex and involves receptors localized in axon terminals, dendritic spines, and glial cells (see Introduction and references therein). Presynaptic mGlu2/3 receptors inhibit glutamate release, and their activation contributes to the induction and expression of long-term depression (LTD) at excitatory synapses (Yokoi et al., 1996; Anwyl, 1999; Huang et al., 1999; Cho et al., 2000; Pöschel and Manahan-Vaughan, 2005; Nicholls et al., 2006; Altinbilek and Manahan-Vaughan, 2009). This mechanism may be relevant to drug addiction, as shown by the evidence that mGlu2/3 receptor-dependent LTD is blunted in the NAc of morphine-abstinent mice (Robbe et al., 2002) and in the striatum of mice chronically exposed to ethanol (Johnson et al., 2020). However, a single exposure to cocaine fails to affect mGlu2/3 receptor-dependent LTD in the NAc (Fourgeaud et al., 2004). A growing body of evidence suggests that postsynaptic mGlu3 receptors interact with mGlu5 receptors to modulate different forms of activity-dependent synaptic plasticity in the PFC and hippocampus (Di Menna et al., 2018; Joffe et al., 2019; Dogra et al., 2021). mGlu3 receptors may also indirectly affect excitatory synaptic transmission by regulating the expression of glutamate transporters in astrocytes (Aronica et al., 2003).

The role of Group 2 mGlu receptors in cognition has been extensively explored, and results are not homogeneous. However, most of the data were obtained in experimental animal models of psychosis in which mGlu2/3 receptor agonists or mGlu2-positive allosteric modulators have consistently produced beneficial effects (Schoepp and Marek, 2002; Swanson and Schoepp, 2003; Hackler et al., 2010; Horiguchi et al., 2011; Griebel et al., 2016; Wischhof and Koch, 2016; Hideshima et al., 2018). In contrast, in normal mice or rats, dual mGlu2/3 receptor antagonists or NAMs induced procognitive effects in different tasks, such as operant conditioning delayed match to position, recognition memory, response inhibition, cognitive flexibility, and spatial learning (Higgins et al., 2004; Shimazaki et al., 2007; Goeldner et al., 2013). Selective mGlu2 NAMs were also found to improve delayed nonmatched to position in mice (Shu et al., 2020) and object retrieval in monkeys (Rudd et al., 2023). Studies carried out in knock-out mice revealed a state-dependent effect of mGlu2 and/or mGlu3 receptors on cognitive function. Double mGlu2/3^−/−^ mice showed an impairment in spatial reference and working memory tasks. However, exposure to stress impaired memory performance in wild-type mice but enhanced performance in mGlu2/3^−/−^ mice (Lyon et al., 2011). mGlu2^−/−^ mice showed impairment in spatial working memory in the elevated T maze, whereas mGlu3^−/−^ mice showed a biphasic effect dependent on the time of training (De Filippis et al., 2015).

Our findings strengthen the hypothesis that the influence of mGlu2 and mGlu3 receptors on cognition is state- and disease-dependent. Genetic deletion of either mGlu2 or mGlu3 receptors did not affect recognition memory in control mice but prevented cognitive dysfunction in methamphetamine-treated mice. Similar findings were obtained with drugs that selectively inhibit either mGlu2 or mGlu3 receptors, with the difference that the mGlu2 receptor NAM, VU6001966, impaired recognition memory in control mice. This contrast with the procogitive effect observed with another selective mGlu2 receptor NAM (a 4-arylquinoline-2-carboxamide derivative) in C57Bl mice challenged with scopolamine (Shu et al., 2020). It is possible that our control CD1 mice developed a paradoxical response to VU6001966 or that VU6001966 had off-target effects that restrained its potential procognitive activity in control mice. This apparent paradox warrants further investigation.

We found that methamphetamine caused a selective increase in mGlu2/3 receptor protein levels in the PFC, a brain region critically involved in recognition memory in the NOR test (Schacter and Slotnick, 2004; Morici et al., 2015; Chao et al., 2022). This increase was not visible in the PFC of both mGlu2 and mGlu3 knock-out mice, suggesting, but not proving, that methamphetamine causes an increased formation and/or stability of mGlu2/3 heterodimers. The density of the immunoreactive band corresponding to mGlu2/3 receptor monomers was also increased in the PFC of wild-type mice after methamphetamine treatment, but this may simply reflect the dissociation of heterodimers caused by the reducing agent present in the sample buffer.

Studies of PFC mGlu2/3 receptor expression in animal models of psychostimulant use disorder produced heterogeneous results. An increase in mGlu2/3 receptor density in the PFC and dorsal striatum was found after cocaine self-administration in Wistar rats (Pomierny-Chamiolo et al., 2017). In contrast, in Long–Evans rats, extended self-administration of methamphetamine caused no changes in PFC mGlu2 receptor levels after 7 d of withdrawal (Hámor et al., 2018) or a reduction in PFC surface mGlu2/3 receptor levels after 14 d of abstinence or forced extinction (Schwendt et al., 2012). No changes in PFC mGlu2/3 protein levels were reported in B6 mice after 10 d of injection of methamphetamine followed by either 1 or 21 d of extinction (Lominac et al., 2016). Thus, changes in PFC mGlu2/3 receptor expression in response to psychostimulants critically depend on the animal species or strain, the regimen of psychostimulant administration, and the time window of drug withdrawal.

In our study, the increase in PFC mGlu2/3 protein levels was not associated with changes in the primary receptor transduction mechanism, i.e., the G_i_-mediated inhibition of adenylyl cyclase activity. This suggests that, after methamphetamine, overexpressed mGlu2/3 heterodimers are less coupled to inhibition of cAMP formation. The upregulation of AGS3 found in the PFC of methamphetamine-treated mice is consistent with this hypothesis. AGS3, which belongs to Class 2 of activators of G-protein signaling, interacts with the GDP-bound α_i_ subunit, biasing G_i_ protein signaling toward βɣ subunit-activated pathways (De Vries et al., 2000; Lanier, 2004). G_βɣ_ subunits activate several effector proteins including G-protein–coupled receptor kinases 2 and 3, Type 2, −4 and −7 adenylyl cyclase, phospholipase Cβ1-3 (Camps et al., 1992; Katz et al., 1992), N- and P/Q-type voltage-sensitive calcium channels (Herlitze et al., 1996; Ikeda, 1996), phostatidylinositol-3-kinase (PI3 K) βɣ, Rac guanine nucleotide exchange factors, and MAP kinase (Smrcka, 2008; Smrcka and Fisher, 2019).

We were surprised to find that activation of mGlu2/3 receptors amplified depolarization-induced [^3^H]-D-aspartate release in synaptosomes prepared from the PFC of methamphetamine-treated mice. To our knowledge, this is the first demonstration of an inverse operation of mGlu2/3 receptors in regulating neurotransmitter release. A bidirectional regulation of glutamate release has also been described for the mGlu7 receptor, a presynaptic mGlu receptor subtype which is also coupled to G_i_ proteins (Martin et al., 2010, 2011, 2018).

The molecular mechanism underlying the inverse operation of mGlu2/3 receptors on glutamate release remains to be identified. Any of the pathways activated by G_βɣ_ subunits might be involved, with MAP kinase, PI3 K, or a direct effect on voltage-sensitive calcium channels being potential candidates. We found that levels of Rab3A and vGlut1, which are established presynaptic markers of glutamatergic transmission, were largely increased in the PFC of methamphetamine-treated mice. Rab3A, a small GTP-binding protein involved in Ca^2+^-dependent exocytosis (Takai et al., 1996), interacts with the Rab-interacting molecule, RIM-1α, in enhancing glutamate release in cortical nerve terminals (Ferrero et al., 2013). Increases in the levels of the vGlut1 might reflect an increased density of glutamate-containing vesicles, an increased density of vGlut1 per vesicle, or sprouting of glutamatergic terminals. Thus, the increase in Rab3A and vGlut1 found in the PFC of methamphetamine-treated mice may help explain the paradoxical enhancing effect of mGlu2/3 receptor activation on [^3^H]-D-aspartate release. Interestingly, changes in Rab3A, vGlut1, and AGS3 caused by methamphetamine treatment were abolished in mice lacking either mGlu2 or mGlu3 receptors, suggesting that (1) an amplifying positive feedback loop exists between AGS3 and mGlu2/3 receptors and (2) endogenous activation of overexpressed mGlu2/3 receptors induces the expression of Rab3A and vGlut1. This hypothesis warrants further investigation.

**Figure 13.**
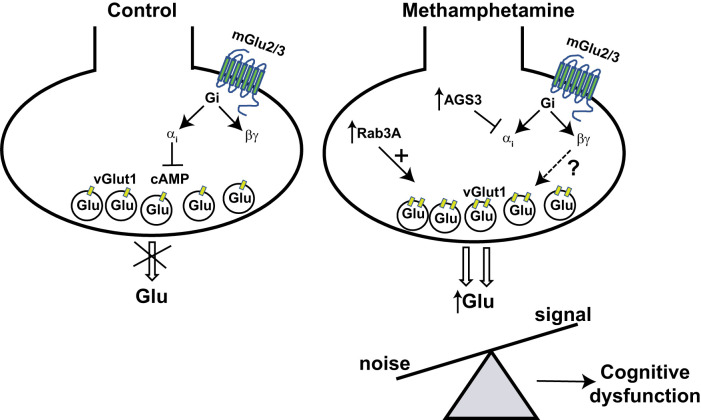
Possible molecular scenario underlying methamphetamine-induced cognitive dysfunction in mice. Treatment with methamphetamine leads to a large increase of expression levels of AGS3, Rab3A, and vGlut-1 in the PFC. The upregulation of AGS3 may cause a bias of G_i_ protein signaling toward βγ subunit-activated pathways. Increased levels of Rab3A may induce an enhanced glutamate (Glu) release in cortical nerve terminals. Higher levels of the Glu vesicular transporter, vGlut1, suggest an increased density of glutamate-containing vesicles or an increased density of vGlut1 per vesicle or sprouting of glutamatergic terminals. Overall, these plastic changes may underlie the paradoxical amplification of Glu release caused by the activation of mGlu2/3 receptors. Therefore, the disruption of one the major mechanisms of the presynaptic regulation of glutamate release in cortical nerve terminals, with ensuing alteration of the signal-to-noise ratio during learning, may be responsible of cognitive dysfunction methamphetamine-induced. *AGS3*, Type 3 activator of G-protein signaling; *Rab3A*, Ras-related protein 3A; *vGlut1*, vesicular glutamate transporter 1.

In conclusion, we have shown that mGlu2 and mGlu3 receptors undergo plastic changes after methamphetamine treatment, and these changes are causally related to cognitive dysfunction, as assessed by the NOR test. These findings provide a remarkable example of how psychostimulants may affect mGlu2/3 receptor function altering the regulation of synaptic activity by these receptors (Fig. 13). The paradoxical amplification of glutamate release caused by the activation of mGlu2/3 receptors after methamphetamine treatment may disrupt one of the major mechanisms of presynaptic regulation of glutamate release, thereby altering the signal-to-noise ratio during learning. An increased synaptic noise caused by the enhanced glutamate release may occlude the signal during memory formation overcoming homeostatic mechanisms, such as synaptic scaling (Turrigiano, 2008), that normally limit the spreading of membrane depolarization to neighboring dendritic spines. In addition, recruitment of multiple neuronal ensembles caused by glutamate spreading outside the synaptic territory may impair mechanisms of pattern discrimination and separation during memory formation. Alterations in postsynaptic mGlu3 receptors might also contribute to dysfunction in NOR memory caused by methamphetamine. We found that mGlu3 receptors lost the ability to boost mGlu5 receptor-mediated PI hydrolysis in PFC slices prepared from methamphetamine-treated mice. This mechanism, which reflects a functional cross-talk between postsynaptic mGlu3 and mGlu5 receptors, regulates different forms of activity-dependent synaptic plasticity in the PFC underlying cognition and responses to stress (Joffe et al., 2019, 2021). Why pharmacological activation of postsynaptic mGlu3 receptors with LY379268 was unable to amplify the PI response to DHPG in methamphetamine-treated mice is unclear. The enhanced glutamate release might have desensitized postsynaptic mGlu3 receptors causing the uncoupling between mGlu3 and mGlu5 receptors. Alternatively, the receptor cross talk might have been occluded by an enhanced mGlu5 receptor signaling, as suggested by the greater PI response to DHPG in slices from methamphetamine-treated mice. Thus, multiple mGlu2/3-mediated adaptive mechanisms taking place both at pre- and postsynaptic levels might be involved in cognitive dysfunction induced by methamphetamine. We suggest that the primary adaptive mechanism involves mGlu2/3 receptors present in presynaptic terminals, which become hyperexpressed and acquire the ability to enhance glutamate release in response to chronic exposure to methamphetamine. Postsynaptic changes in mGlu3 receptor signaling and mGlu3–mGlu5 receptor cross talk might be secondary to changes in glutamate release, but this remains to be demonstrated.

We used male mice in our study, and we cannot predict how mGlu2/3 receptors behave in female mice challenged with methamphetamine. Female sex hormones are known to interact with mGlu2/3 receptors at multiple levels (Boulware et al., 2005; Chaban et al., 2011; Wong et al., 2019), and their fluctuation during the ovarian cycle might critically affect mGlu2/3 responses to methamphetamine. This variable is difficult to control with the paradigm of methamphetamine treatment we have used in the present study.

We suggest that mGlu2, mGlu3, or mGlu2/3 receptor NAMs (which can block either homo- or heterodimers) could be potential candidates for the treatment of cognitive dysfunction in individuals affected by MUD. The choice of the specific drug depends on how the drug affects the cycle of methamphetamine addiction, i.e., the phase of binge, withdrawal, and prolonged vulnerability to relapse, considering that the impact of mGlu2 and mGlu3 receptors on these processes can be strictly region-dependent and that plastic changes of receptor function in our study were restricted to the PFC.

## Synthesis

Reviewing Editor: Nathalie Ginovart, University of Geneva Decisions are customarily a result of the Reviewing Editor and the peer reviewers coming together and discussing their recommendations until a consensus is reached. When revisions are invited, a fact-based synthesis statement explaining their decision and outlining what is needed to prepare a revision will be listed below. The following reviewer(s) agreed to reveal their identity: NONE. Note: If this manuscript was transferred from JNeurosci and a decision was made to accept the manuscript without peer review, a brief statement to this effect will instead be what is listed below.

Both reviewers agreed that this manuscript provides interesting data with respect to the role of mGlu2/3 receptors in the memory deficits induced by methamphetamine exposure. Both reviewers also raised some concerns that need to be addressed in a revision before the paper can be accepted.

Please find the reviewer's comments below:

Reviewer 1:

Busceti et al. present a series of studies describing how methamphetamine exposure affects mGluR function within the prefrontal cortex. A 5-day treatment with methamphetamine impaired novel object recognition and increased levels of mGlu2/3 within PFC. The behavioral changes were not present in mGlu2 or mGlu3 knockout and were blocked by administration of selective NAMs. Further mechanistic studies showed intact regulation of cAMP but described interesting and novel changes to signaling molecules related to presynaptic release machinery. Methamphetamine treatment reversed the directionality of mGlu2/3 regulation, such that activation of mGlu2/3 promoted aspartate release from synaptosomes. This was associated with upregulation of AGS3, Rab3A, and vGluT1. The studies are logical and well-controlled, and provide an interesting lead on potential treatments for methamphetamine use disorder, a disease without any available pharmacological treatments. Nonetheless, there are some concerns that reduce enthusiasm for the manuscript in its present form

Major concerns

1. cAMP formation in KO mice appears to have a lower sample size than that from WT mice. This seems susceptible to a false-negative result. Also, did the authors assess for genotypic differences in cAMP levels (basal and/or FSK-stimulated)? It looks like mGlu2 KO may have higher levels and mGlu3 KO may have lower. If so, it seems possible that a floor or ceiling effect could be contributing to the negative results. To that point, it would be nice to see the pharmacological conditions in the aspartate release experiment to get a sense of the contributions of mGlu2 vs mGlu3 in control mice, since there are some developmental compensations that occur in the KO mice

2. Do the authors have any thoughts or data regarding the cellular localization of the methamphetamine-induced upregulation of mGlu2/3 (pre/post synaptic or glial?) or the upregulation of AGS3? Also, assuming it is presynaptic - which terminals (local or extra-cortical afferents) might be expressing receptors? If available, cell type-specific analysis (either IHC or RNA in situ hybridization) would enhance the interpretation of the current findings.

Minor concerns

1. Data in Fig 1 should be first analyzed with a two-way ANOVA. Once an interaction is reported, then post-hoc tests can be used to identify differences between groups. I don't expect this analysis will change the interpretation. Similar issue with Fig 4, in that a two-way ANOVA should be first performed to test for main effect of methamphetamine and interactions

2. Control mice treated with VU6001966 did not show novel object recognition (Fig 1E). This is briefly mentioned in the Discussion but I would like to see additional thoughts on this matter, particularly considering the cited literature that mGlu2 NAMs can improve certain aspects of cognition in mice

3. It would be helpful for Abstract to include some background information containing the overall scope of the problem. The abstract also states 10 mg/kg methamphetamine, which I believe is an error

4. The word "abusers" in significance statement. This term carries a pejorative connotation and I would suggest replacing with "individuals/persons who use methamphetamine" or just striking "in abusers" from the sentence entirely.

5. Line 87 - would be helpful to include some transitions or additional language to highlight the disconnect between the authors' recent KO studies (where mGlu2 KO showed reduced DA release and CPP) versus previous pharmacological studies (where mGlu2 PAMs/agonists reduce drug-seeking)

6. Some discussion is warranted as to whether the authors expect or don't expect that a similar effect would be observed in females

7. Please specify whether experiments were conducted in the light or dark phase

8. It appears that methamphetamine animals have slightly increased familiar exploration time along with decreased novel exploration time. This might suggest deficits are specific to recognition memory, with novelty-seeking being unaffected. Is this consistent with prior studies?

9. WB analysis in WT mice seems to show that the dimer bands are reduced to a greater extent in mGlu3 KO than in mGlu2 KO. There are many reasons this could be the case, and I think this deserves some additional discussion/speculation. First, this could indicate that mGlu3 receptors have a greater propensity to form heterodimers than mGlu2 receptors (see Lee et al. 2020 for related analysis). Alternatively, it is possible that mGlu2 heterodimers with other receptors (e.g., mGlu4) are present at significant levels. Finally, it is possible that mGlu3 homodimers are more stable and resistant to reducing conditions than mGlu2 homodimers.

10. Along these lines, The authors speculate that mGlu2/3 heterodimers are the main receptor population that is upregulated by methamphetamine treatment. Yet, previous studies using the same pharmacological tools suggest that the mGlu2 NAM, but not the mGlu3 NAM, increases glutamate release probability in PFC (Joffe et al. 2020). Doesn't this suggest that either (1) mGlu2 homomers are the major presynaptic receptor, or (2) VU0650786 does not inhibit mGlu2/3 heterodimers?

11. Discussion (329-330) includes instances where prior drug (morphine and alcohol) exposure reduces mGlu2/3-LTD in striatum. However, this discussion should also incorporate data indicating that a single exposure to cocaine (which has a mechanism more similar to methamphetamine than the morephine/alcohol) does not affect mGlu2/3-LTD in NAC (Fourgeaud et al 2004)

12. Second paragraph of discussion should incorporate the current findings. Do the authors think that AGS3 signaling could underlie the interaction between mGlu2/3 antagonism and stress on working memory?

Reviewer 2 :

The present manuscript by Busceti et al. aims to investigate the role of mGlu2 and mGlu3 glutamate receptors in methamphetamine-induced recognition memory deficits using a knockout mouse model and various ex-vivo and in vivo pharmacological approaches. The rationale for the conducted research is strong, as it is well-documented that prolonged exposure to methamphetamine produces memory deficits in human users and experimental animals. Still, the neurobiological mechanisms of methamphetamine-induced behavioral deficits are incompletely understood. The main finding is that methamphetamine-induced deficit in recognition memory in the NOR task coincides with upregulation of mGlu2/3 in the prefrontal cortex and that constitutive and brain-wide deletion of mGlu2 or mGlu3 (or acute systemic inhibition of these receptors) prevents this deficit. The authors then aimed to identify synaptic mechanisms that might be responsible, though uncovered somewhat paradoxical findings regarding the control of mGlu2/3 over glutamate/aspartate release and reported discrete changes in synaptosomal proteins in methamphetamine- vs saline-treated animals. Overall, the study provides new information regarding the role of mGlu2/3 in post-methamphetamine cognitive deficits, though it does not directly address the relationship between observed deficit and motivation to seek the drug and fails to consider cellular and regional diversity of mGlu2/3 function. The reviewer has the following specific concerns:

Major concerns:

1) The studies do not consider the diversity of mGlu2 vs 3 regional and cellular distribution, which might affect the interpretation of the data

a. For example, in the PFC, mGlu3 receptors (at least in primates, with some evidence from rodents) are mainly located on the glia and on postsynaptic elements, while mGlu2 are predominately presynaptic. Accordingly, studies by the Arnsten lab at Yale suggested that mGlu3 ACTIVATION (likely acting via a postsynaptic mechanism) is pro-cognitive, while mGlu2 activation lacks a clear pro-cognitive effect. How do authors reconcile these data with their behavioral and ex vivo findings?

b. The authors introduce brain-wide mGlu2/3 manipulations (genetic or NAMs) but then interpret their findings exclusively through PFC-dependent mechanisms. Lesion and disconnection studies by the Warburton lab showed that PFC activity is not necessary for NOR memory. On the other hand, the perirhinal cortex is necessary for NOR memory, but it is omitted from the current study (e.g. see; PMID: 17360918). Is it possible that relevant mGlu2/3 adaptations take place in the perirhinal cortex?

c. In relation to the comment above, none of the interventions used in the study are brain-region- or cell-type-specific.

2) The study omits female rats. This is a critical omission, as sex differences in glutamate transmission (and receptor expression) in the PFC have been reported. Also, studies by the Mermelstein lab documented sex/estrogen-dependent changes in mGlu2/3 receptors and the distinct contribution of this receptor to drug-seeking depending on sex and estrogen levels.

3) What was the timing of tissue collection relative to the NOR test? Is it possible that the handling of animals and/or the test itself affected the biological data?

Minor concerns:

1) The authors report detecting mGlu receptor monomers and dimers but do not specify the strength or the type of reducing agents. The specifics of reducing conditions are known to affect the relative amounts of dimers vs. monomers detected by immunoblotting.

2) Missing citation and a typo: Ln 357-358.

"Similar findings were obatined with drugs that selectively inhibit either mGlu2 or mGlu3 receptors, with the difference that the mGlu2 receptor NAM, VU6001966, impaired recognition memory in control mice."

3) The study reported a somewhat paradoxical finding that "LY379268, amplified

depolarization-induced [3H]-D-aspartate release in superfused synaptosomes prepared from the PFC of methamphetamine-treated mice, " Was this effect tested across a range of LY doses or just at a single dose? Include a justification of the single dose.

4) It seems that the NOR retention session was conducted 24 hours after the training session, but this is not specified in the manuscript.

## Author Response

We have prepared a point by point response to the reviewer's comments. In the following text, reviewer's comments are reported as RC and highlighted in italics, our answers as AC in plain text. We have performed new experiments to address the kind issues raised by the Reviewers, as indicated.

Reviewer #1 - Major concerns RC 1. cAMP formation in KO mice appears to have a lower sample size than that from WT mice. This seems susceptible to a false-negative result. Also, did the authors assess for genotypic differences in cAMP levels (basal and/or FSK-stimulated)? It looks like mGlu2 KO may have higher levels and mGlu3 KO may have lower. If so, it seems possible that a floor or ceiling effect could be contributing to the negative results. To that point, it would be nice to see the pharmacological conditions in the aspartate release experiment to get a sense of the contributions of mGlu2 vs mGlu3 in control mice, since there are some developmental compensations that occur in the KO mice.

AC: We have implemented the sample size in cAMP experiments with mGlu2 and mGlu3 KO mice (see new Fig. 9B-C). If there is a ceiling effect, this is in wild-type mice, in which the extent of stimulation with forskolin is greater than in the two KOs. Nonetheless, LY379268 was able to significantly reduce cAMP in wild-type mice treated with saline or methamphetamine. Absolute levels of cAMP in response to forskolin are similar in slices from mGlu2 KO mice, but the extent of stimulation is lower than in wild-type mice because basal levels of cAMP are higher in mGlu2 KO mice. Nonetheless, there is no effect of LY379268 on cAMP. The extent of stimulation with forskolin is the lowest in mGlu3 KO mice, and, in spite of this, there is no effect of LY379268. We therefore believe that our data are now solid and suggest that (i) methamphetamine treatment does not alter the inhibition of adenylyl cyclase resulting from mGlu2/3 receptor activation in the PFC; and, (ii) both mGlu2 and mGlu3 receptors are involved in this particular response.

RC 2. Do the authors have any thoughts or data regarding the cellular localization of the methamphetamine-induced upregulation of mGlu2/3 (pre/post synaptic or glial?) or the upregulation of AGS3? Also, assuming it is presynaptic - which terminals (local or extra-cortical afferents) might be expressing receptors? If available, cell type-specific analysis (either IHC or RNA in situ hybridization) would enhance the interpretation of the current findings.

AC: This is an important comment, and we are aware that the lack of data on cellular localization may limit the relevance of our findings. However, conventional IHC does not help to address this comment because of the high background using the mGlu2/3 antibody (for example, mGlu3 receptors are present not only in neurons but also in astrocytes and microglia). This could be addressed with quantitative EM analysis with immunogold but this is far beyond our technical skills. We could perform a number of lesions of afferent pathways in an attempt to localize the precise localization of up-regulated mGlu2/3 receptors. This, however, can be addressed in a new long project with new permission for animal research. However, we moved from our previous findings showing that postsynaptic mGlu3 receptors (mGlu2 receptors are exclusively presynaptic to our knowledge) are functionally linked to mGlu5 receptors, and their activation boost mGlu5 receptor-mediated PI hydrolysis (Di Menna et al., Neuropharmacology, 2018). Interestingly, this particular form of receptor-receptor interaction occurs in the PFC and is involved in different forms of synaptic plasticity in physiology and pathology (Joffe et al., Neuropharmacology, 2019). We performed new experiments measuring the PI response to the mGlu1/5 agonist, DHPG, alone or combined with the group-II mGlu agonist, LY379268 in PFC slices prepared from animals treated with saline or methamphetamine. Interestingly, in slices treated with methamphetamine LY379268 failed to potentiate the PI response to DHPG. Thus, a dysfunction of postsynaptic mGlu3 receptors (or at least in the cross-talk between postsynaptic mGlu3 and mGlu5 receptors) might have a role in methamphetamine-induced cognitive dysfunction. New data are shown in Fig. 9D,D', presented in the Results sections at pag. 13, lines 319-321 and pag. 14, lines 322-325 and discussed at pag. 19, lines 469,470 and pag. 20, lines 471-476. Methods are described at pag. 9, lines 215-221.

Reviewer #1 Minor concerns RC 1. Data in Fig 1 should be first analyzed with a two-way ANOVA. Once an interaction is reported, then post-hoc tests can be used to identify differences between groups. I don't expect this analysis will change the interpretation. Similar issue with Fig 4, in that a two-way ANOVA should be first performed to test for main effect of methamphetamine and interactions.

AC: Thanks for the comment. Statistical analysis was performed with parametric tests only for data showing a normal distribution for all groups. When even just one group of data did not follow a normal distribution, we used non-parametric tests. Normal distribution was assessed by the D'Agostino &Pearson test.

Data in the Figure 1 (new Figure 2) and Figure 4 (new Figure 10) were analyzed as follows:

- Figure 2C: data for all groups showed a normal distribution and were analyzed by two-way ANOVA for repeated measures (familiar vs novel) with the Bonferroni's post-hoc test for multiple comparison;

- Figure 2E-saline and 2F-methamphetamine: data showed a normal distribution and were analyzed by parametric paired two-tailed Student's t-test (familiar vs novel);

- Figure 10A-saline; 10B-saline; 10C-methamphetamine: data showed a normal distribution and were analyzed by one-way ANOVA followed by the Bonferroni's test for multiple comparisons;

- Figure 2A; 2B; 2D; 2E-methamphetamine; 2F-saline: data did not follow a normal distribution and were analyzed by Wilcoxon two-tailed test for paired values (familiar vs novel);

- Figure 10A-methamphetamine; 10B-methamphetamine; 10C-saline: data did not follow a normal distribution and were analyzed by Kruskal-Wallis test followed by the Dunn's test for comparison across multiple groups.

We feel this was the right approach.

RC 2. Control mice treated with VU6001966 did not show novel object recognition (Fig 1E). This is briefly mentioned in the Discussion but I would like to see additional thoughts on this matter, particularly considering the cited literature that mGlu2 NAMs can improve certain aspects of cognition in mice.

AC: Yes indeed. We were the first to be surprised. We have now offered some possible explanations at pag. 17, lines 399-403.

3. It would be helpful for Abstract to include some background information containing the overall scope of the problem. The abstract also states 10 mg/kg methamphetamine, which I believe is an error.

AC: The Abstract has been re-written accordingly.

RC 4. The word "abusers" in significance statement. This term carries a pejorative connotation and I would suggest replacing with "individuals/persons who use methamphetamine" or just striking "in abusers" from the sentence entirely.

AC: Yes, sorry for that. The word "abusers" has been replaced with "individuals who use methamphetamine" RC 5. Line 87 - would be helpful to include some transitions or additional language to highlight the disconnect between the authors' recent KO studies (where mGlu2 KO showed reduced DA release and CPP) versus previous pharmacological studies (where mGlu2 PAMs/agonists reduce drug-seeking).

AC: We have now highlighted that our previous findings on mGlu2/3 receptors and methamphetamine are not consistent with other studies on alcohol, cocaine or nicotine seeking (see pag. 4, lines 96-98).

RC 6. Some discussion is warranted as to whether the authors expect or don't expect that a similar effect would be observed in females.

AC: We cannot predict how mGlu2/3 receptors behave in mice challenged with methamphetamine because female sex hormones interact with mGlu2/3 receptors at multiple levels. This is now discussed in the last paragraph of pag. 20, lines 477-482.

RC 7. Please specify whether experiments were conducted in the light or dark phase.

AC: We have specified in the section Methods of the revised manuscript that all experiments were conducted in the light phase. ¬¬ RC 8. It appears that methamphetamine animals have slightly increased familiar exploration time along with decreased novel exploration time. This might suggest deficits are specific to recognition memory, with novelty-seeking being unaffected. Is this consistent with prior studies? AC: Not to our knowledge.

RC 9. WB analysis in WT mice seems to show that the dimer bands are reduced to a greater extent in mGlu3 KO than in mGlu2 KO. There are many reasons this could be the case, and I think this deserves some additional discussion/speculation. First, this could indicate that mGlu3 receptors have a greater propensity to form heterodimers than mGlu2 receptors (see Lee et al. 2020 for related analysis). Alternatively, it is possible that mGlu2 heterodimers with other receptors (e.g., mGlu4) are present at significant levels. Finally, it is possible that mGlu3 homodimers are more stable and resistant to reducing conditions than mGlu2 homodimers.

AC: This is absolutely correct. However, this difference emerges in the lanes loaded with tissue from mGlu2 and mGlu3 KO mice used to control the size of the bands (for example, see the last two lanes in the representative WB in Fig. 4A). Discussing the nature of potential heterodimers (including the mGlu2-mGlu4 heterodimer) is beyond the scope of this manuscript.

RC 10. Along these lines, the authors speculate that mGlu2/3 heterodimers are the main receptor population that is upregulated by methamphetamine treatment. Yet, previous studies using the same pharmacological tools suggest that the mGlu2 NAM, but not the mGlu3 NAM, increases glutamate release probability in PFC (Joffe et al. 2020). Doesn't this suggest that either (1) mGlu2 homomers are the major presynaptic receptor, or (2) VU0650786 does not inhibit mGlu2/3 heterodimers? AC: We mentioned the possibility that methamphetamine enhances mGlu2/mGlu3 heterodimer levels because the increase was abolished in either mGlu2 or mGlu3 KO mice. We consider unlikely that mGlu2 homomer are up-regulated by methamphetamine, because, if so, we should have still observed an up-regulation in the PFC of mGlu3 KO mice in response to methamphetamine (and vice versa for mGlu3 homomers in the PFC of mGlu2 KO mice).

RC 11. Discussion (329-330) includes instances where prior drug (morphine and alcohol) exposure reduces mGlu2/3-LTD in striatum. However, this discussion should also incorporate data indicating that a single exposure to cocaine (which has a mechanism more similar to methamphetamine than the morephine/alcohol) does not affect mGlu2/3-LTD in NAC (Fourgeaud et al 2004).

AC: Thanks for the suggestion. This is now included in the first paragraph of the discussion pag. 15 line 371 and pag. 16, line 372.

RC 12. Second paragraph of discussion should incorporate the current findings. Do the authors think that AGS3 signaling could underlie the interaction between mGlu2/3 antagonism and stress on working memory? AC: We cannot speculate on the role of AGS3 on the interaction between mGlu2/3 antagonism ad stress on working memory on the basis of our findings. It will be an interesting future project.

Reviewer #2 - Major concerns RC 1. The studies do not consider the diversity of mGlu2 vs 3 regional and cellular distribution, which might affect the interpretation of the data. a. For example, in the PFC, mGlu3 receptors (at least in primates, with some evidence from rodents) are mainly located on the glia and on postsynaptic elements, while mGlu2 are predominately presynaptic. Accordingly, studies by the Arnsten lab at Yale suggested that mGlu3 ACTIVATION (likely acting via a postsynaptic mechanism) is pro-cognitive, while mGlu2 activation lacks a clear pro-cognitive effect. How do authors reconcile these data with their behavioral and ex vivo findings? b. The authors introduce brain-wide mGlu2/3 manipulations (genetic or NAMs) but then interpret their findings exclusively through PFC-dependent mechanisms. Lesion and disconnection studies by the Warburton lab showed that PFC activity is not necessary for NOR memory. On the other hand, the perirhinal cortex is necessary for NOR memory, but it is omitted from the current study (e.g. see; PMID: 17360918). Is it possible that relevant mGlu2/3 adaptations take place in the perirhinal cortex? c. In relation to the comment above, none of the interventions used in the study are brain-region- or cell-type-specific.

AC a. We have performed new experiments examining the ability of the mGlu2/3 agonist to boost mGlu5-mediated PI hydrolysis in PFC slices prepared from mice treated with saline or methamphetamine. This response (Di Menna et al., 2018) is mediated by postsynaptic mGlu3 receptors and the functional cross-talk between mGlu3 and mGlu5 receptors is involved in the regulation of synaptic plasticity in the PFC (Joffe et al., 2019; 2021; all quoted in the manuscript). We found that amplification of DHPG-stimulated PI hydrolysis by LY379268 (a response exclusively mediated by mGlu3 receptors - Di Menna et al., 2018) is lost in PFC slices prepared from methamphetamine-treated mice. This might contribute to cognitive dysfunction. Data are presented in the new Fig. 9D,D', presented in the Results sections at pag. 13 lines 319-321 and pag. 14, lines 322-325, and discussed at pag. 19, lines 469,470 and pag. 20, lines 471-476. Methods are described at pag. 9, lines 215-221.

AC b. We have been extended the analysis of mGlu2/3 receptor expression in the perirhinal cortex. The analysis was performed by immunohistochemistry because the amount of tissue was not sufficient for WB analysis. mGlu2/3 receptor expression was unchanged in response to methamphetamine in the three genotypes. Data are shown in the new Fig. 8, and described in the Results section at pag. 13, lines 311-314. Methods are reported at pag. 11, lines 251-265.

AC c. Yes, we are aware. A nice way will be to use light-sensitive drugs activated in brain regions that are relevant to methamphetamine addiction. This will be a nice follow-up of the manuscript.

RC 2. The study omits female rats. This is a critical omission, as sex differences in glutamate transmission (and receptor expression) in the PFC have been reported. Also, studies by the Mermelstein lab documented sex/estrogen-dependent changes in mGlu2/3 receptors and the distinct contribution of this receptor to drug-seeking depending on sex and estrogen levels.

AC: Yes, we agree. We are aware that estrogens interact with mGlu2/3 receptors at multiple levels. Using females we should consider the impact of the different phases of the ovarian cycle and extend the study to a large number of animals. This is why we have limited the study to males. All this is now discussed at pag. 20, lines 477-482.

RC 3. What was the timing of tissue collection relative to the NOR test? Is it possible that the handling of animals and/or the test itself affected the biological data? AC: Mice were killed 4 hours after the retention phase of the NOR test. The handling of mice did not affect western blot analysis as shown by new data obtained in saline- or methamphetamine-treated mice which had not been exposed to behavioral tests (see new Fig. 7). This is also reported in the Results section at pag. 13, lines 309,310.

Reviewer #2 - Minor concerns RC 1. The authors report detecting mGlu receptor monomers and dimers but do not specify the strength or the type of reducing agents. The specifics of reducing conditions are known to affect the relative amounts of dimers vs. monomers detected by immunoblotting.

AC: We have now specified in the section Methods that western blot analysis was performed by loading proteins in a sample buffer containing 10% 2-mercaptoethanol.

RC 2. Missing citation and a typo: Ln 357-358. "Similar findings were obatined with drugs that selectively inhibit either mGlu2 or mGlu3 receptors, with the difference that the mGlu2 receptor NAM, VU6001966, impaired recognition memory in control mice." AC: Typo has been corrected RC 3. The study reported a somewhat paradoxical finding that "LY379268, amplified depolarization-induced [3H]-D-aspartate release in superfused synaptosomes prepared from the PFC of methamphetamine-treated mice, " Was this effect tested across a range of LY doses or just at a single dose? Include a justification of the single dose.

AC: The study was conducted by using two concentrations of LY379268 (1 nM and 10 nM) (see Figure 10).

RC 4. It seems that the NOR retention session was conducted 24 hours after the training session, but this is not specified in the manuscript.

AC: Yes, correct. This is now included in the Methods section at pag. 7.

We are grateful to both reviewers for their kind comments, which helped us to improve the manuscript.

With my best regards.

Sincerely, Ferdinando Nicoletti
